# Lateral Flow Assays for Pathogen Detection: Reporting Mechanisms and Applications

**DOI:** 10.3390/biology15211980

**Published:** 2026-11-02

**Authors:** Teng Qin, Xiaochen Wang, Xingyao Shen, Lei Ye, Qingqing Dou, Jinliang Liu

**Affiliations:** 1 School of Environmental and Chemical Engineering, Shanghai University, Shanghai 200444, China; 2 Shandong Center for Food and Drug Evaluation & Inspection, Ji’nan 250014, China; 3 Department of Biomedical Engineering, The University of Alabama at Birmingham, Birmingham, AL 35255, USA

**Keywords:** lateral-flow assay (LFA), reporting markers, pathogen detection

## Abstract

Pathogens present significant risks to public health and typically require detection in a hospital or clinic setting. The advancement of lateral flow assay (LFA) cassettes has made detection accessible to all, allowing individuals to perform tests from nearly any location. The extensive use of antigen rapid test (ART) kits for self-screening of COVID has reinforced the critical role of LFAs in accelerating diagnostic processes and subsequent disease prevention measures. This study delves into the realm of lateral flow assays, encompassing their fundamental design principles, interpretation of results, and the cutting-edge research focused on enhancing reporter materials and signal amplification techniques. Additionally, it emphasizes recent progress in employing LFAs for the effective identification of a diverse array of pathogenic microorganisms. This comprehensive overview has the potential to serve as a valuable reference for researchers in related disciplines.

## Simple Summary

Lateral flow assays (LFAs) are widely used diagnostic tools due to their speed and ease of use, yet traditional versions often lack the sensitivity required for early disease detection. This review explores the next generation of LFAs, which utilize advanced nanomaterials—such as fluorescent, magnetic, and enzymatic nanoparticles—to significantly boost detection limits. We examine how specific physical characteristics, including particle size, shape, and surface charge, directly dictate the performance of these tests. Furthermore, we provide a critical assessment of the current barriers preventing these high-performance assays from reaching the market, specifically highlighting the difficulties in mass manufacturing, batch-to-batch reproducibility, and long-term stability. The review concludes that while the technology is promising, future efforts must focus on solving these engineering challenges to ensure reliable, cost-effective point-of-care diagnostics.

## 1. Introduction

Test strips have become a widely utilized method for diagnosing COVID throughout the pandemic [1,2]. With a single swab of body fluid, Antigen Rapid Test (ART) kits can provide accurate results within a timeframe of 5 to 10 min. These kits, categorized as a type of Point-of-Care Testing (POCT), necessitate minimal training, resulting in decreased manpower requirements in comparison to Polymerase Chain Reaction (PCR) tests. Moreover, they are associated with considerably lower costs and offer a much quicker turnaround than PCR methodologies, while also being less constrained by the testing environment. Test strips enhance convenience for both healthcare providers and patients. This tool serves as a valuable supplement in situations where precise testing methods, such as PCR instruments, are unavailable. Additionally, it has the potential to minimize nosocomial transmission by facilitating triage while awaiting PCR test results.

The ART operates on the principles of lateral flow assays (LFAs), which are paper-based biosensing platforms that analyze liquid samples as they move through the test via capillary action. The results are visually depicted with high sensitivity, allowing for straightforward interpretation through intuitive outcomes [3,4]. Initial iterations of LFAs primarily provide qualitative or semi-quantitative outcomes, which are adequate for detecting analytes present in significantly higher concentrations than ordinary levels. Conventional test strips utilize AuNPs as indicators, with the human chorionic gonadotropin (HCG) test for pregnancy serving as a prominent example, which was established in 1980s. Subsequently, commercial lateral flow tests (LFTs) have been developed to screen various infectious diseases, including human immunodeficiency virus (HIV), influenza A/B virus, group A *Streptococcus* and malaria. These LFAs are crucial for the prevention of transmissible diseases and contribute significantly to public health efforts globally.

However, as the need for testing low-concentration analytes has increased, particularly in complex human bodily fluids, the efficacy of AuNPs has proven inadequate for yielding reliable results. Consequently, numerous advancements have been made to enhance the sensitivity of LFAs, including modifications to traditional reporters, the development of new reporters and the implementation of signal amplification technologies. Novel labeling agents have been developed as alternatives to AuNPs to improve the detection sensitivity of analytes present in lower concentrations, thereby providing additional options for analysis. Prominent examples of these agents include magnetic nanoparticles (MNPs) [5,6], colored particles [7,8], upconversion nanoparticles (UCNPs) [9,10], quantum dots (QDs) [11,12,13,14], and various other fluorescent nanoparticles [15,16].

LFAs can be engineered to detect various analytes, contingent upon the identification of specific recognition molecules. A variety of pathogen recognition molecules have been utilized in LFAs, encompassing nucleic acids [17], aptamers [18], proteins [19,20,21], and traditional antibodies used in the immunoassays. In this context, numerous signal amplification methods have been explored alongside LFAs, such as surface-enhanced Raman scattering (SERS) [5,22,23], PCR [24,25], Loop-mediated isothermal amplification (LAMP) [26], thermal contrast amplification [27], among others. As a result, LFAs can be applied to a diverse array of complex samples, including serum [28], whole blood [29], urine [30], saliva [31], sweat [32], and other body fluids [33]. Nevertheless, the intricate nature of these samples poses significant challenges for the labeling agents and detection methodologies used.

In this Review, we provide an overview of the principles and outcome evaluation methods of LFTs. We have examined key parameters essential for assessing the accuracy, repeatability, and overall quality of these tests. The evolution of reporting techniques and the role of signal enhancement have also been covered, with illustrative examples provided throughout the discussion. The review emphasizes the detection of a variety of pathogens, offering key insights and lessons from current practices. It also elaborates on the detection of bacteria with specific examples, highlighting the practical applications of LFTs in diagnostic processes. A particular focus has been placed on the most recent innovations that have emerged since 2020, reflecting the rapid progress and adaptation of LFT technology in response to emerging health challenges, such as the COVID pandemic. By concentrating on recent developments, this review aims to provide a snapshot of the current state and future potential of LFTs, particularly in the context of POC testing, where rapid and accurate diagnostic capabilities are crucial.

## 2. Lateral Flow Assays (LFAs)

LFA Test strips are specifically designed to identify characteristic receptors of pathogens, thereby facilitating the detection of diseases. When antibodies are employed as the recognition elements, this approach is referred to as lateral flow immunoassays (LFIAs). Additionally, nucleic acids and proteins can also serve as recognition sites, though they typically require integration with amplification techniques such as PCR or Loop-Mediated Isothermal Amplification (LAMP) to enhance sensitivity. The fundamental principle of a lateral flow assay (LFA) involves the capillary-driven flow of a liquid sample along a test strip. A pretreated liquid sample is applied to the test strip, which is composed of a polymeric porous cellulose substrate. The classic LFIA architecture is not a monolithic substrate but a modular assembly of distinct functional components mounted on a backing card. The fluid dynamics and assay performance rely on the specific properties of each layer:

- Sample Pad: Typically cellulose or glass fiber, treated to ensure uniform sample delivery.
- Conjugate Pad: Usually glass fiber, responsible for storing and releasing the dried biorecognition conjugates.
- Nitrocellulose (NC) Membrane: The reaction matrix where the specific capture lines are immobilized.
- Absorbent Pad: Cellulose fiber acting as a fluid sink to drive capillary flow.
- Backing Card: The structural support (typically PVC) holding these components in alignment.

As the sample flows through the strip, it passes over immobilized recognition molecules. Upon binding to these molecules, a visible or detectable signal is generated, indicating the presence or concentration of the target analyte in the sample.

### 2.1. Structure and Test Principle

**Figure 1:**
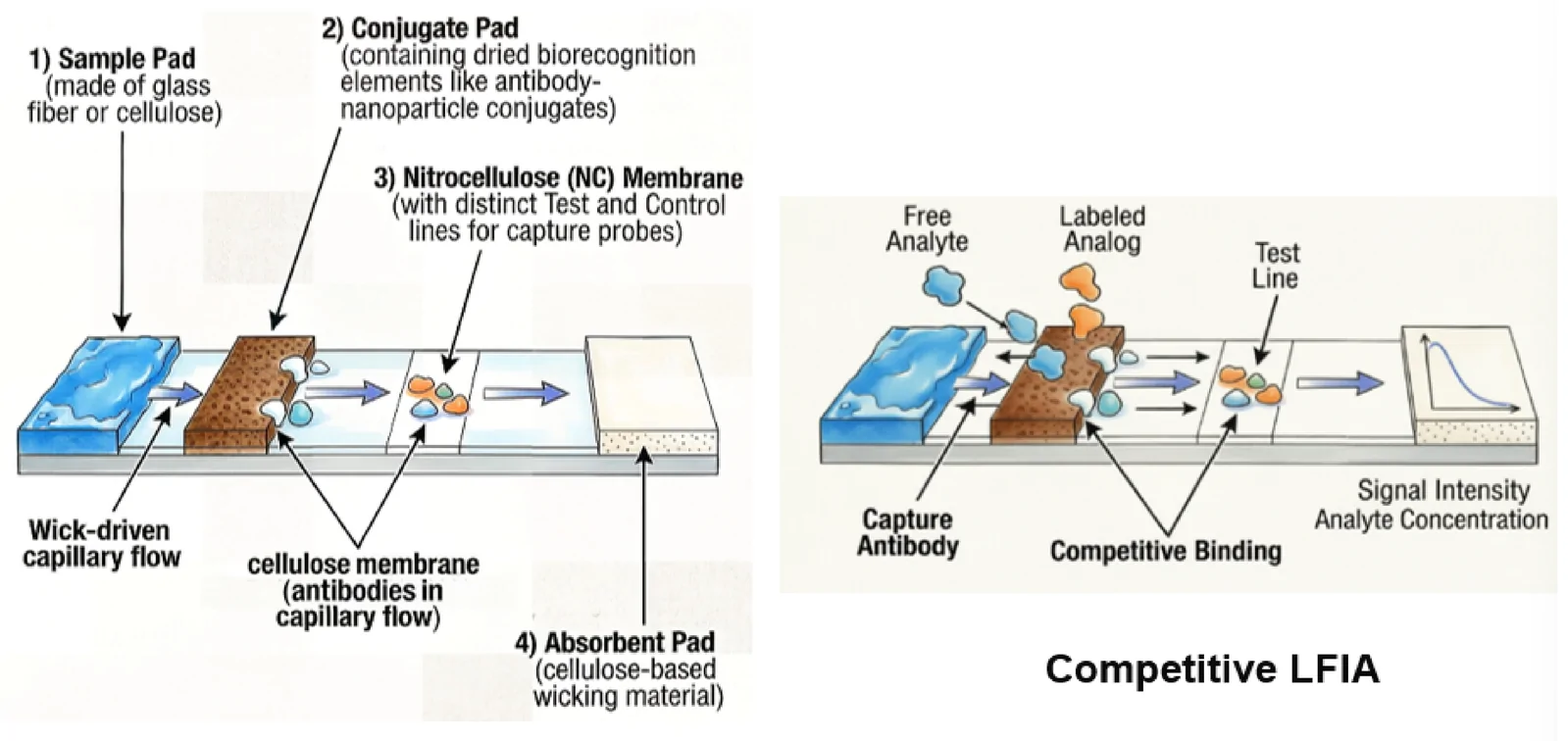
Schematic representation of ordinary setup of a lateral flow immunoassay.

Lateral Flow Assays (LFA) are membrane-based diagnostic platforms designed to detect the presence of a specific target analyte in a liquid sample. The fundamental principle relies on the specific interaction between the target analyte and a complementary biorecognition element. It is crucial to distinguish between the analyte and the recognition element: Target Analytes are the substances being detected (e.g., pathogen antigens, nucleic acids, or antibodies). Biorecognition Elements are the specific probes (e.g., antibodies, aptamers, or oligonucleotide probes) that bind to the target. The lateral flow test strip typically consists of five crucial components: sample pads composed of glass fiber, a nitrocellulose membrane with a fixed test line (T line) and control line (C line), a conjugate release pad containing bioreceptors (antibodies or aptamers) specific to the pathogen, an absorption pad, and a support pad to facilitate the assembly of all the components (Figure 1). The sample pad is infused with buffer salts and surfactants, which facilitate the sample’s compatibility with the detection system. The sample pad guarantees that the target analyte within the sample can effectively bind to the bioreceptor on the membrane. The conjugate release pad is equipped with a colored or fluorescent reporter-labeled specific bioreceptor designed to target analytes. The analyte, bound to the recognition agent and reporter, migrates with the liquid and will ultimately be captured by the test line, provided that the target analyte is present. Thus, the emergence of the reporter signal at the test line indicates a positive result. The control line typically comprises secondary receptors specific to the marker-labeled receptor used in the conjugate pad. The appearance of a signal at the control line confirms proper fluid flow and validates the assay’s operation. Consistent signal appearance in both test line and control line is imperative. If the signal is observed only at the test line, it indicates a potential issue with the assay’s performance, such as improper liquid flow. In such cases, it is recommended to repeat the test to ensure accurate results.

The majority of LFAs function based on a comparable fundamental mechanism. In this section, we elucidate the principles of testing associated with LFIAs to provide a comprehensive understanding of the testing process. Most commercially available assays utilize two distinct types of antibodies or other recognition molecules, resulting in the formation of a sandwich structure, wherein both antibodies engage with the analyte. The primary antibody is fixed onto the test line in test strip, while the secondary antibody is labeled with a detectable marker and stored in the conjugate release pad. Upon loading a sample onto the strip, any present antigen will initially be captured by the secondary antibody on the release pad. During the movement of the liquid sample, the antigen–antibody-marker complex will be captured by the immobilized primary antibody, resulting in the formation of a sandwich structure at the test line. The antigen can subsequently be quantified by assessing the signal emitted by the markers. This setup is ideal for large-size analytes that contain multiple antigenic sites. For instance, the human chorionic gonadotropin (HCG) used in pregnancy tests features multiple antigenic sites, allowing two specific antibodies to simultaneously target HCG [34]. Similarly, the p24 antigen from the HIV, which is a large viral core protein [35], can also be analyzed with this direct assay method. The use of two antibodies guarantees the recognition of only the appropriate antigen, thereby improving the specificity of the signal and minimizing the chances of non-specific interactions. Nevertheless, the employment of two antibodies raises the overall expense of the test strip.

Molecules that are small or possess only a single antigenic determinant cannot engage in simultaneous binding with two antibodies [36]. To overcome this limitation, competitive assays are utilized. In the most common competitive format, a labeled analyte conjugate (the analyte or its analog conjugated to a detectable marker such as AuNPs) is dried on the conjugate pad and serves as the migrating component. The test line is pre-immobilized with a fixed amount of capture antibody specific to the analyte. The control line is coated with a secondary antibody that binds the labeled conjugate, ensuring assay validity. When a sample is applied, the liquid rehydrates the labeled analyte conjugate on the conjugate pad, and both the conjugate and any free analyte present in the sample migrate together toward the test line. At the test line, the free analyte and the labeled analyte conjugate compete for the limited binding sites of the immobilized capture antibody. If the analyte concentration in the sample is low or absent, the labeled conjugate binds to the capture antibody, producing a strong visible signal at the test line. Conversely, if the analyte concentration is high, the free analyte saturates the antibody binding sites, preventing the labeled conjugate from binding, resulting in a weak or absent signal at the test line. Thus, in this configuration, the test-line signal intensity is inversely proportional to the analyte concentration in the sample. This is in direct contrast to the sandwich format, where signal intensity increases with analyte concentration.

The competitive format simplifies the assay by requiring only one analyte-specific antibody and one labeled analyte conjugate, as opposed to the two analyte-specific antibodies needed in sandwich assays. However, it should be noted that while competitive LFAs may offer advantages for detecting small molecules with single epitopes, the cost difference compared to sandwich assays is highly dependent on the specific reagents and conjugation chemistries employed. Additionally, the analytical sensitivity of competitive LFAs can be limited when detecting analytes at very low concentrations, and the potential for cross-reactivity of the labeled conjugate with structurally similar molecules may affect specificity.

### 2.2. Test Result Assessment

#### 2.2.1. Analytical Performance: Limit of Detection (LOD)

The analytical performance of an LFA is primarily characterized by its Limit of Detection (LOD), which represents the lowest concentration of the target analyte that can be reliably distinguished from a blank sample (i.e., the background noise) with a specified level of statistical confidence, typically 95%. For LFAs, the LOD is empirically established through serial dilution of a known concentration of the target analyte in a defined matrix (e.g., buffer, serum, or other biological fluid). The lowest concentration at which the test line signal is statistically distinguishable from the signal of a negative control—based on the mean signal of the blank plus three standard deviations (mean_blank_ + 3σ)—is designated as the LOD.

It is important to distinguish between visual LOD and instrument-read LOD. The visual LOD refers to the lowest analyte concentration at which a test line can be discerned by the naked eye and is inherently subjective, varying between observers and lighting conditions. In contrast, instrument-read LOD relies on a dedicated reader (e.g., a fluorescence scanner, smartphone-based imaging system, or SERS detector) to quantify the signal intensity, thereby enabling more objective and often substantially lower detection thresholds. For example, while AuNP-based visual LFAs typically achieve LODs in the ng/mL to μg/mL range, fluorescent or SERS-based LFAs with instrument readout can reach pg/mL levels (as detailed in Section 3). The LOD achieved is therefore a function of both the reporter chemistry and the readout modality, and comparisons across studies should account for these variables.

#### 2.2.2. Clinical Diagnostic Performance: Sensitivity and Specificity

**Figure 2:**
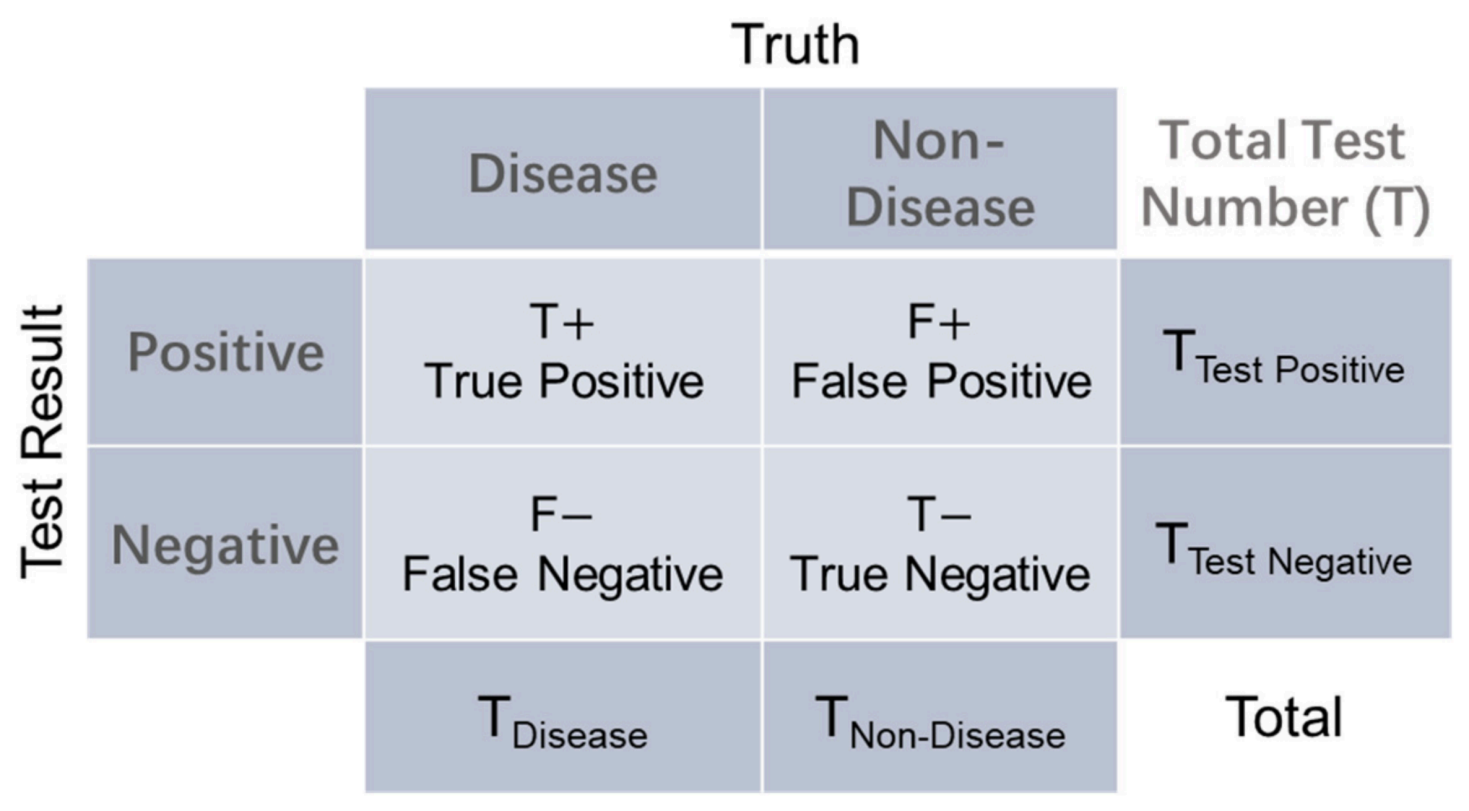
Schematic representation of diagnostic results for a disease.

Distinct from analytical LOD, the clinical diagnostic performance of an LFA is evaluated using clinical sensitivity and clinical specificity, which reflect the test’s accuracy when applied to patient populations. Clinical sensitivity (also termed diagnostic sensitivity) is defined as the proportion of individuals with the target disease who yield a positive test result [True Positives/(True Positives + False Negatives)]. Clinical specificity (diagnostic specificity) is defined as the proportion of individuals without the disease who yield a negative test result [True Negatives/(True Negatives + False Positives)]. These metrics are determined by comparing the LFA results against an established reference standard (gold standard), typically across a cohort of clinically characterized patient samples, and are summarized in a 2 × 2 contingency table (Figure 2).

#### 2.2.3. Relationship Between Analytical LOD and Clinical Performance

A critical distinction that warrants emphasis is that an improvement in analytical LOD does not necessarily translate into improved clinical sensitivity in patient testing. Several factors contribute to this disconnect:

Matrix effects: An LOD established in simple buffer may not reflect detection capability in complex clinical specimens (e.g., whole blood, serum, or nasal swab eluates), where interfering substances such as proteins, lipids, or heterophilic antibodies can suppress signal or cause nonspecific binding.

Target accessibility: In clinical samples, the analyte may exist in forms (e.g., complexed with host antibodies, aggregated, or partially degraded) that differ from the purified reference material used for LOD determination, potentially reducing the effective concentration available for capture.

Disease-stage dependence: The concentration of the target biomarker varies with disease stage; an assay with an excellent LOD may still miss early or late infections if the biomarker level falls below the detection threshold during those phases.

Sample size and prevalence: Estimates of clinical sensitivity and specificity are subject to statistical uncertainty, particularly in small cohorts, and are influenced by disease prevalence in the tested population.

Therefore, while a lower analytical LOD is a desirable and necessary feature of an improved LFA, it should be viewed as a prerequisite rather than a guarantee of superior clinical diagnostic performance. Robust clinical validation using well-characterized patient samples remains essential to establish the real-world utility of any LFA. Throughout this review, we use “analytical sensitivity” or “LOD” when referring to the detection limit of the assay and reserve “clinical sensitivity” for performance in patient testing, in order to avoid ambiguity.

**Sensitivity** = number of true positives/(number of true positives + number of false negatives)

**Specificity** = number of true negatives/(number of true negatives + number of false positives)

While the choice of reporter significantly influences the assay’s sensitivity—defined here by the Signal-to-Noise Ratio (S/N) and Limit of Detection (LOD)—the biochemical specificity of the platform is fundamentally determined by the recognition elements. In this study, high specificity is achieved through the use of high-affinity monoclonal antibodies with minimal cross-reactivity, combined with a rigorous blocking strategy to mitigate matrix effects. The instrumental readout serves to accurately quantify the specific binding events by maximizing the signal intensity relative to the background noise, rather than altering the inherent selectivity of the immunoreaction.

### 2.3. Innovation and Alteration of Lateral Flow in Test Strips

The fundamental principles of lateral flow test strips are well established. However, traditional fluid flow theories and models still exhibit significant limitations when applied in practice. Firstly, current LFT design often hinges on simplified assumptions in fluid flow [37], depicting flow as one-dimensional and uniform, while overlooking the intricate multi-dimensional flow dynamics and intricate interactions between materials and fluids. These oversimplifications can lead to significant deviations between the predicted results and the actual flow patterns, as well as the material-fluid interactions observed in real detection processes. For instance, under real conditions, fluid flow is influenced by the surface properties of materials such as sample pads, conjugate pads, and nitrocellulose (NC) membranes, and may exhibit non-uniform distribution or localized retention of the fluid, posing challenges in achieving precise control over its movement. Secondly, traditional flow models fail to fully account for the dynamic changes in fluid flow, such as variations in sample concentration gradients, changes in fluid viscosity, and the impact of external environmental factors (such as temperature and humidity) on flow behavior [38]. These factors can lead to instability in fluid flow during actual detection, thereby affecting the repeatability and accuracy of the detection results. Moreover, conventional models fall short in comprehensively capturing the intricacies of fluid-material interactions, thereby failing to precisely depict fluid dynamics within porous media. Consequently, the intricate processes of permeation and diffusion of fluids through such media remain insufficiently explored.

Therefore, the behavior of fluid flow plays a pivotal role in determining the performance of LFTS. For instance, variations in fluid flow velocities can lead to insufficient mixing of the sample and reagent, potentially impacting the reliability of the test results. Moreover, the intricate interaction between a fluid and a material surface, influenced by factors such as surface hydrophilicity, hydrophobicity, and charge distribution, significantly influences the dynamics of their interplay. By optimizing the structural design of microchannels with specific sizes and shapes, the efficiency of fluid mixing can be enhanced, and non-specific binding can be reduced. Consequently, this improves the accuracy and stability of detection in LFTS, ensuring more reliable test results. For instance, the Unisart StructSure^®^ has implemented a wave-like structural flow pattern in LFA [39]. The channel bends induce Dean flows (secondary flow vortices) and increase the interfacial surface area between fluid streams, which enhances mixing via diffusion and transverse transport within the laminar regime.

**Figure 3:**
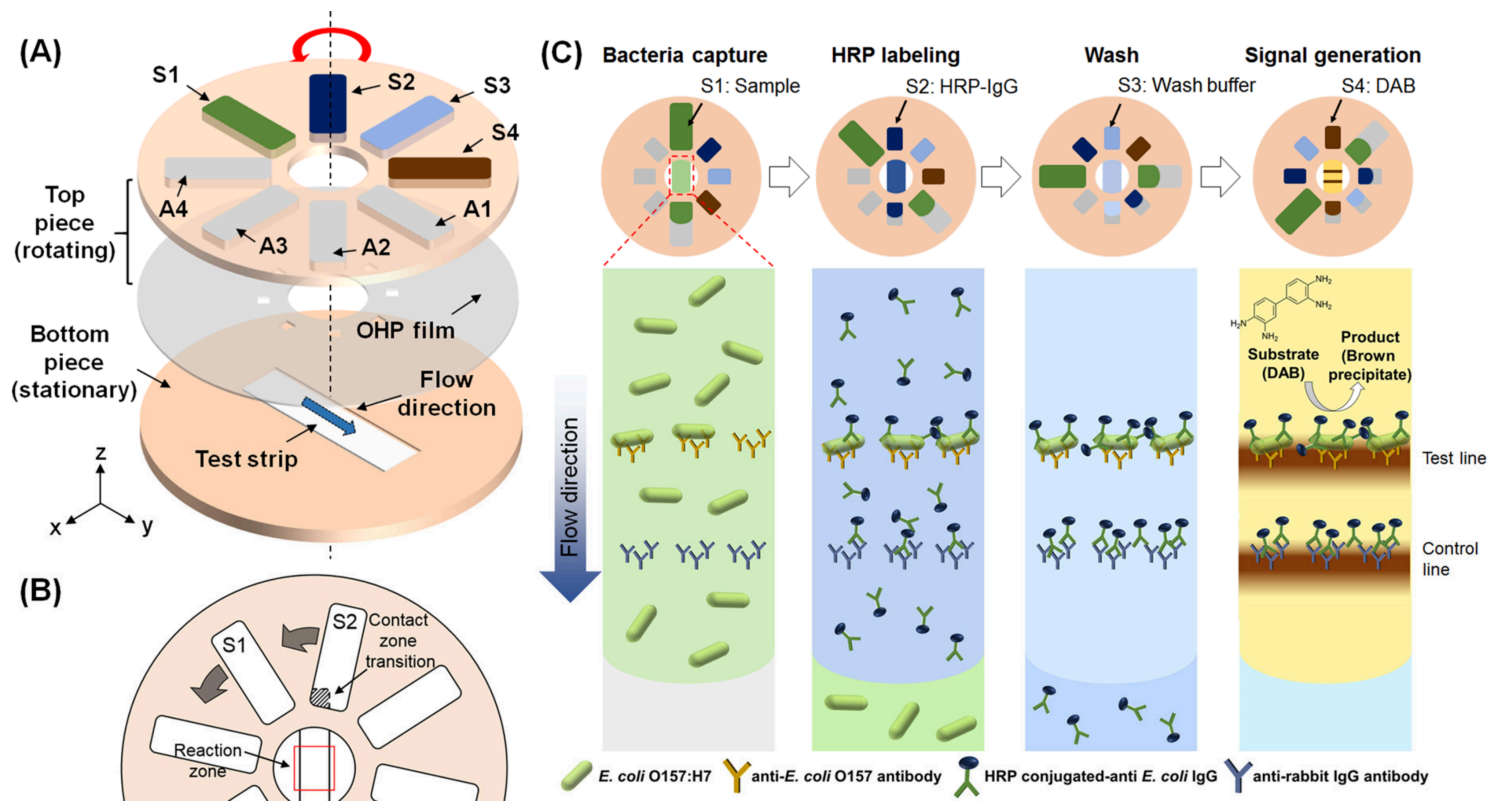
(**A**) Layout and design of a rotary lateral flow test device, optimized for the precise delivery of multiple reagents and execution of multistep assays. (**B**) Elutriation of the top view, highlighting the dynamic transition of the contact zone as the device rotates. (**C**) Schematics of the step-by-step process of strip-based ELISA, encompassing bacteria capture, HRP-IgG labeling, washing, and signal generation steps. Reprint permission from Analytical Chemistry 2016 [40].

Another structurally innovative design involves integrating the assay channel with a rotary device [40]. In this configuration, the test strip remains stationary while the top portion of the device rotates, aligning the reagent and absorbent pads with each end of the paper strip and initiating fluid flow (Figure 3). Through further incremental rotation, an adjacent pair of pads aligns simultaneously, facilitating the flow of subsequent reagents that were preloaded in the reagent pad. This device empowers users to regulate the sequence and duration of fluid flow, thereby streamlining the process of dispensing multiple reagents onto the strip. This design facilitates the effortless delivery of multiple reagents to a paper strip, thereby supporting complex multistep assays.

Later on, more advanced rotary designs have emerged in the literature. For instance, Ji et al. introduced innovative rotational paper-based microfluidic chips capable of detecting phenolic pollutants [41]. The chip is able to perform both qualitative and quantitative analyses of 4-nitrophenol (4-NP) and 2,4,6-trinitrophenol (TNP) concurrently, thus demonstrating its efficacy in simultaneous detection of these compounds. Pattarapon et al. created an automated rotary lateral flow immunoassay platform that incorporates a servo motor [42]. This multistep immunoassay is capable of quantifying the colorimetric detection of E. coli O157:H7 bacteria. A three-dimensional (3D) microfluidic analytical device was developed to conduct multistep diagnostic assays in limited space. This device enables dual colorimetric and electrochemical detection, exhibiting a wide dynamic range for detection, with a remarkable detection limit of 0.35 ng/mL for cardiac Troponin I [43].

These innovative structural designs clearly demonstrate significant benefits. Nevertheless, the processes involved in preparation and manufacturing can be rather complex, which may result in increased costs and longer lead times. This complexity might also influence the yield of qualified products. Consequently, the primary challenge is to uphold cost-effectiveness while guaranteeing production feasibility, without sacrificing either element.

## 3. Markers/Labels Used in LFA

A comprehensive understanding of LFA technology requires not only a description of the diverse detection modalities reported in the literature but also a structured framework that enables meaningful comparison across studies. A well-recognized challenge in the field is that individual publications often focus on a single aspect of the LFA architecture—for instance, a novel nanoparticle reporter, an improved signal amplification strategy, or a portable reader device—making it difficult to situate each contribution within the broader technology landscape. Without a common reference framework, comparisons risk becoming category errors: a study optimizing gold nanoparticle synthesis (reporter level) cannot be directly benchmarked against a study improving smartphone-based image analysis (reader level), as they address fundamentally different engineering questions.

**Figure 4:**
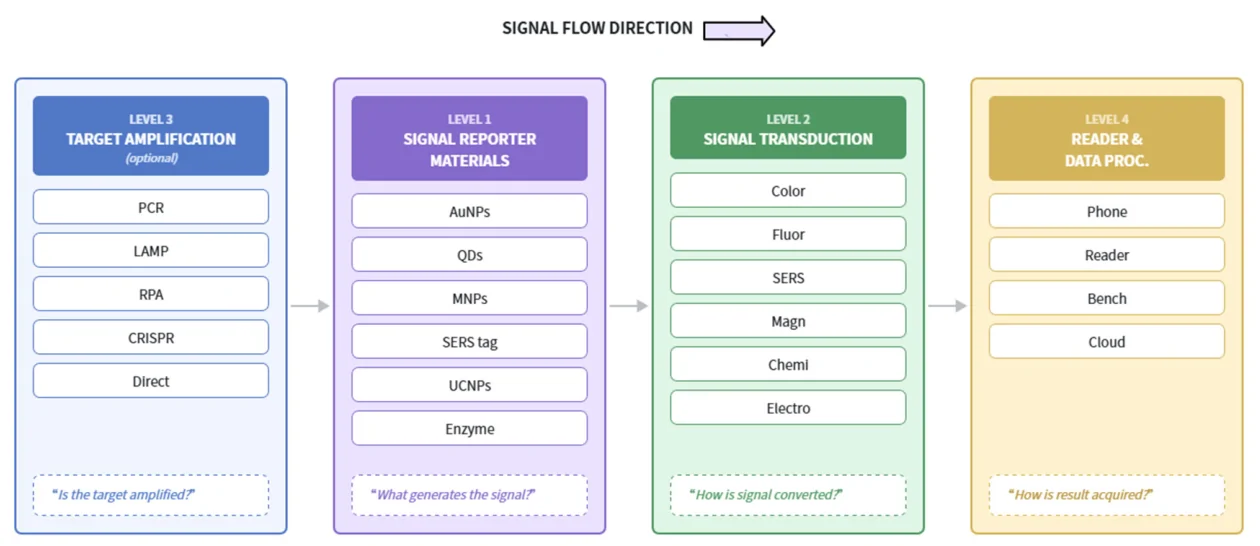
Four-Level Architecture of Lateral Flow Assay Technology.

To address this challenge, we introduce a four-level hierarchical framework that deconstructs any LFA system into its constituent functional modules. Drawing on the architectural logic of the LFA workflow, we define four distinct levels, each corresponding to a stage in the signal generation and acquisition chain (Figure 4):

Level 1—Signal Reporter Materials: The physicochemical entity that generates the detectable signal at the test and control lines. This encompasses the full diversity of reporter technologies: metallic nanoparticles (AuNPs, AgNPs), quantum dots (QDs), upconversion nanoparticles (UCNPs), magnetic nanoparticles (MNPs), SERS nanotags, carbon nanoparticles, fluorescent dyes, chemiluminescent probes, electrochemical labels, and enzyme-based reporters.
Level 2—Signal Transduction and Readout Modality: The physical principle by which the reporter signal is converted into a measurable output. This includes colorimetric absorbance (visual or instrumented), fluorescence, surface-enhanced Raman scattering (SERS), magnetic detection, chemiluminescence, electrochemistry, and thermal/photothermal detection.
Level 3—Target Amplification (optional): Whether and how the target analyte is amplified before detection. This includes nucleic acid amplification methods (PCR, LAMP, RPA, NASBA), CRISPR-Cas-based signal amplification, rolling circle amplification (RCA), as well as direct detection without amplification.
Level 4—Reader and Data Processing Platform: The hardware and software system that acquires, quantifies, and interprets the transduced signal. This ranges from naked-eye visual inspection and smartphone cameras to dedicated portable readers, benchtop instruments, and cloud-connected or AI-assisted data processing pipelines.

Importantly, these four levels are orthogonal to the choice of detection mode. That is, both equipment-free and instrument-dependent LFAs can be decomposed into the same four-level architecture—the distinction lies in which technologies populate each level, not in the framework itself. As we shall demonstrate, this cross-cutting framework enables rigorous within-level comparisons (e.g., comparing AuNPs vs. carbon nanoparticles, both at Level 1) while preventing spurious cross-level comparisons (e.g., comparing a novel reporter (Level 1) to a novel reader (Level 4) as if they were competing alternatives).

With this analytical framework established, we now proceed to the primary classification of LFA technologies. Following the convention most relevant to end-users—and consistent with the WHO ASSURED criteria for point-of-care diagnostics— we organize the discussion around two major categories based on detection mode: (I) Naked-Eye Detection, where the test result is directly visible to the user without any external instrumentation, and (II) Instrument-Assisted Detection, where an external reader or signal acquisition device is required.

Lateral flow strip tests rely on markers to indicate the presence or absence of a target pathogen. These markers fall into two main categories based on how they convey their signals: visual labels [44], which provide a direct and readily observable color change that can be read by the naked eye, and labels that require specialized instruments for detection [45]. These visual labels are commonly employed in rapid diagnostic tests due to their convenience and immediate results, whereas the labels requiring instrumental detection often offer higher sensitivity and specificity but involve a more complex analytical process.

When a material demonstrates observable changes, such as variations in color, fluorescence, or precipitation, upon interacting with the analyte, it can function as a visual marker in LFA. Nonetheless, the efficacy of these visual markers is generally limited to scenarios where significant concentrations of analytes are present in the samples. The process of visual assessment has inherent constraints. To overcome these limitations, the implementation of advanced LFT labels is essential for enhancing both sensitivity and specificity. In instances where low concentrations of analytes are analyzed or quantified, it is customary to use labels in combination with specialized instruments to ensure accurate detection. Instruments such as fluorescence microscopes [46], flow cytometers, spectrophotometers, chemiluminescence detectors [47,48], autoradiography instruments, or electrochemical analyzers [49,50] are typically utilized for effective detection and quantification. These instrumental labels offer higher sensitivity and accuracy compared to visual labels, making them ideal for precise quantification and high-throughput pathogen detection in laboratory settings.

### 3.1. Visible Markers

Visual markers are now seamlessly integrated into LFIA strip technology in pathogen detection [51]. This integration enables rapid, cost-effective, and user-friendly identification of diverse pathogens [52], a crucial feature in applications ranging from medical diagnostics to food safety [53,54] and environmental monitoring [55,56]. With this visual labeling technology, on-site testing and preliminary screening become highly accessible, providing an efficient and dependable solution for scenarios demanding immediate and accurate results.

#### 3.1.1. Gold Nanoparticle (AuNPs)

Gold nanoparticles (AuNPs) serve as one of the most prevalent visual markers in LFTs [57] owing to their distinctive optical attributes and straightforward synthesis process. AuNPs exhibit vibrant color as a result of surface plasmon resonance (SPR), a phenomenon that occurs when the conduction electrons in the metal nanoparticles collectively oscillate in response to the incoming light. The SPR property enables the AuNP to exhibit size-dependent colors (such as red, blue, and purple) by manipulating the particle size or aggregation state [58]. Typically, AuNPs with a 13 nm diameter turn out wine-red, while an increase in size results in a red shift. When AuNPs aggregate at distances shorter than their diameter, they exhibit a blue color due to interparticle plasmon coupling. The robust plasmonic signal of AuNPs allows for visualization with the naked eye, making them a compelling option for markers in LFT. Additionally, AuNPs can be easily conjugated with biomolecules, including antibodies and aptamers, enhancing their versatility for various pathogen diagnostics. The synthesis of colloidal AuNPs is relatively straightforward, accommodating large-scale production needs and consequently reducing the costs associated with LFA. There are several stability considerations for AuNP-based LFAs. Gold nanoparticles themselves, as inorganic colloids, are inherently resistant to thermal degradation and do not undergo the photobleaching that limits organic fluorophores. However, this does not mean that AuNP-based LFAs are intrinsically stable under heat and humidity. The functional stability of an AuNP-LFA depends on at least three interacting factors:

(1) Conjugate integrity. The antibody adsorbed or covalently conjugated to the AuNP surface is typically the most labile component. Exposure to elevated temperatures (e.g., >40 °C) can induce partial denaturation of surface-immobilized antibodies, reducing antigen-binding capacity and potentially causing conjugate aggregation through exposure of hydrophobic residues. Humidity accelerates this process by plasticizing the protein hydration shell and facilitating conformational mobility.
(2) Test strip formulation. Stabilizers incorporated into the conjugate pad (e.g., trehalose, sucrose, BSA, PVP, PEG) and the nitrocellulose membrane (e.g., surfactant coatings, blocking agents) play a decisive role in determining overall strip stability. A well-formulated strip with optimized stabilizer composition can maintain >90% of initial signal intensity after 12 months at ambient temperature, whereas a poorly formulated strip with identical AuNP-antibody conjugate may lose >50% activity within weeks.
(3) Packaging and storage conditions. Desiccated packaging with humidity indicators and oxygen scavengers can extend shelf life substantially, but improperly sealed pouches allow moisture ingress that accelerates conjugate degradation and alters membrane flow characteristics.

Rather than asserting that AuNPs are ‘stable’, it is more accurate to state that AuNP-based LFAs, when properly formulated with optimized stabilizers, adequate antibody loading, and hermetic packaging, can achieve ambient-temperature shelf lives of 12–24 months—a key advantage over fluorescent labels that may require cold-chain storage. However, this stability is a property of the formulated product, not of the AuNP core alone.

Despite these advantages, the use of AuNPs in LFA is limited by their low sensitivity, due to the fact that naked-eye detection is only reliable when pathogens are present at high concentrations [59]. Ongoing efforts aim to enhance and optimize AuNP tags to overcome these limitations. For example, red and blue dual-color AuNPs were used in one LFA to enhance the sensitivity of tests [59]. Spherical red AuNPs were used to label control line and durian-shaped blue AuNPs were used to label the test line, respectively. The utilization of distinct colors for the two lines significantly improved both the diagnostic sensitivity and specificity, achieving 100% when detecting human Brucella-positive serum, even at a dilution factor of 10^−5^. The LOD was recorded at 0.04 IU/mL, which is two orders of magnitude lower than traditional LFIA strips. A study was conducted by Byzova et al. to investigate the impact of AuNP shape on the LOD [60]. In their study, two types of AuNPs were compared as labels in LFIA for the detection of the cardiac marker troponin I. One type was quasispherical AuNPs with average diameters ranging from 18.6 to 47.5 nm, while the other type was superspherical AuNPs with average sizes of 20.2 to 90.4 nm. Their findings demonstrated that the superspherical AuNPs achieved an 8-fold reduction in LOD when contrasted with the quasispherical AuNPs. Due to the minute interparticle gaps, which facilitate profound LSPR hybridization, nanoclusters exhibit a remarkable augmentation in plasmonic scattering, stemming from the intricate plasmon coupling processes. In the context of LFA, AuNP dimers have been employed with the intent of elevating their operational efficacy [61]. The detection of porcine epidemic diarrhea virus in clinical samples achieved 50 times greater sensitivity than that of a test utilizing spherical colloidal gold. Another study showed that the AuNP aggregates, induced by polyamidoamine (PAMAM) dendrimers, improved the sensitivity up to five times for the aptamer-based LFA in rapid detection of ochratoxin A [62]. In addition to designing novel AuNPs, integrating them with other labels represents a strategic approach to enhance the signals in LFTs.

Due to surface plasmon resonance, Au and Ag nanoparticles could produce significantly amplified electromagnetic fields near their surface with an enhancement factor up to 10^14^ [63]. This resonance effect significantly amplifies molecular vibration in the vicinity of the Au or Ag nanoparticles, resulting in inelastic light scattering [64]. Employing SERS spectroscopy enables the identification of inelastic light scattering from the molecules, characterized by a spectrum with a narrow full width at half maximum, thereby generating highly sensitive signals [65]. Consequently, the integration of SERS technology with LFA presents a novel approach for highly sensitive detection. In recent years, SERS-based LFAs have been effectively implemented for precise and quantitative analysis of diverse targets. However, the low chemical stability of Au or Ag nanotags necessitates engineering modifications before their practical application.

#### 3.1.2. Colloidal Carbon

Colloidal carbon is also a widely employed marker in LFT. This is attributable to several key advantages, including its exceptional stability, minimal cost, and the simplicity with which it can be produced on a large scale [66]. Colloidal carbon particles form black or gray visible signals on the strip, serving as an effective visual indicator. Shi et al. [67] employed colloidal carbon as a marker in a test strip designed to detect antibodies against *Mycoplasma bovis*, a pathogen that could cause severe respiratory diseases, otitis media and mastitis in cattle and tuberculosis in humans as well. The strips demonstrated remarkable performance with 100% specificity and 97.67% sensitivity, with 99.49% concordance with the ELISA test. The high specificity and sensitivity of the LFT offer veterinarians and farmers a user-friendly tool to promptly identify and control outbreaks of *M. bovis*, thereby enhancing disease management in livestock. Colloidal carbon was employed in the competitive LIFA test strip designed to detect the fungal pathogen *Botrytis cinerea* [68], notorious for causing gray mold disease in herbal medicine plants. This test strip showcased remarkable sensitivity, achieving a limit of detection (LOD) of 6.25 μg/mL specifically for identifying IgG3 and IgG2a antibodies. The significance of this lies in its ability to facilitate early detection, preventing the rapid progression of the disease by up to four days, thereby averting substantial economic losses in the herbal medicine industry.

#### 3.1.3. Polymer Microbeads and Nanoparticles

**Figure 5:**
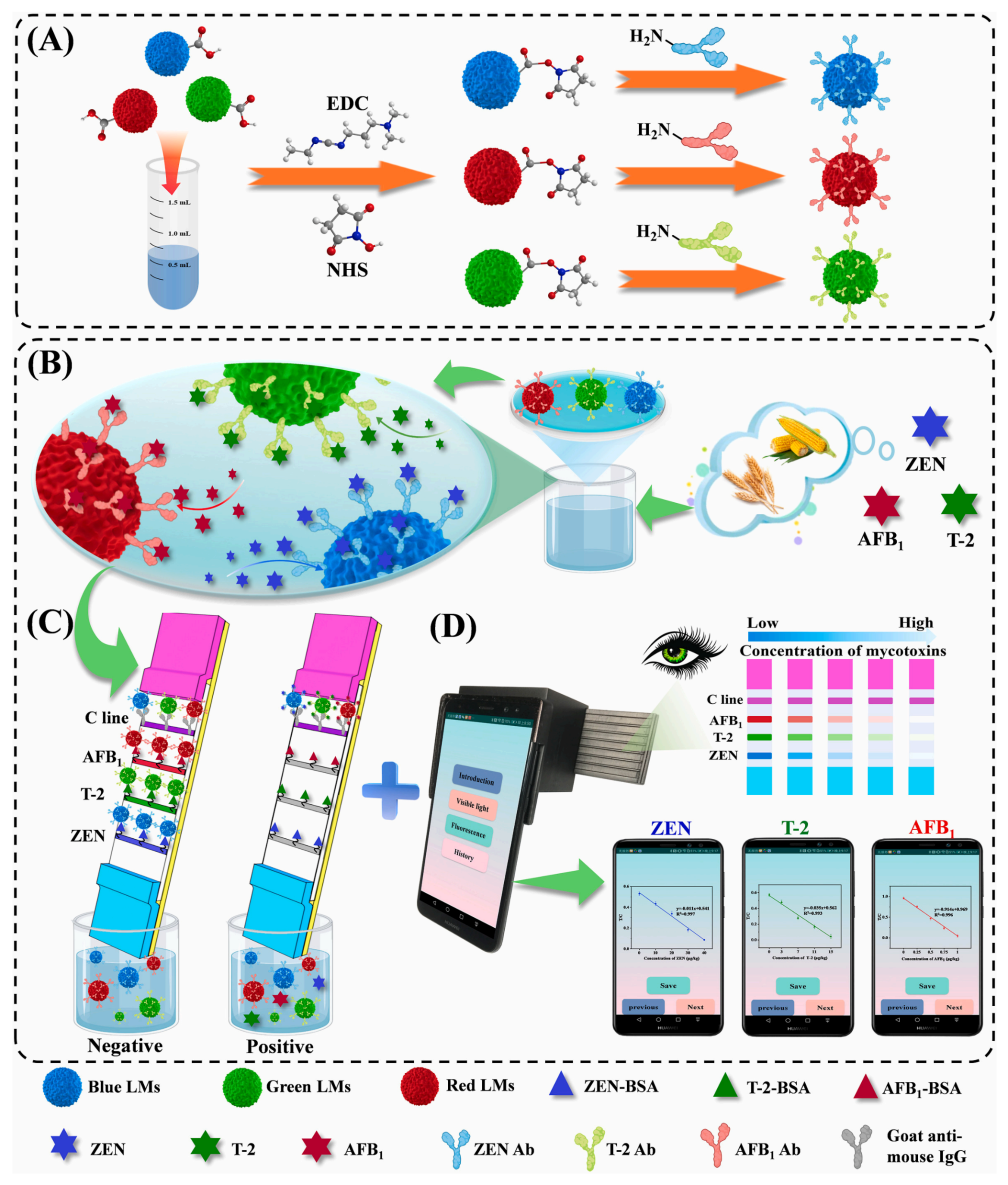
Principles of preparation and specific identification of analytes for the three kinds of LMs-Abs probes, structure of rainbow latex microspheres lateral flow immunoassay (LMs-LFIA) and detection principles of test strips, and qualitative and quantitative test results. (**A**) The method to prepare color bead tags; (**B**,**C**) The principle and structure of test strips; (**D**) The qualitative and quantitative results for the test of analytes. Reprinted (adapted) with permission from Ref. [70]. Copyright 2022 Analytica Chimica Acta.

Polymer nanoparticles and microspheres exhibit distinct advantages in lateral flow assays owing to their ability to be tailored and endowed with unique physicochemical properties. For instance, polymer nanoparticles possessing excellent biocompatibility, such as those made from polystyrene and polylactic acid, provide stability and functionality in intricate biological environments. Moreover, polymer beads can be intricately designed to incorporate a core–shell structure or a porous architecture, with the goal of enhancing their dye loading capacities and signal amplification efficiency. Surface properties can be tailored to boost interaction efficiency with the target analyte, thereby improving their uniformity and stability in lateral flow tests. Latex microbeads are versatile colloidal particles that have been widely used in lateral flow tests owing to their user-friendliness, cost-effectiveness, and capacity to deliver rapid, visible results. These microbeads were among the pioneering materials employed as labels in lateral flow diagnostic tests for qualitative assay or semi-quantification [69]. Traffic light colored red, green, and blue latex microspheres were fabricated in an LFT to identify aflatoxin B1 (AFB1), T-2 toxins (T-2), and zearalenone (ZEN) in cereals, respectively (Figure 5) [70]. The LOD and limit of quantitation (LOQ) of the LFIA with these latex labels could be as low as 0.04/0.40/1.21 and 0.09/0.99/1.56 μg kg^−1^ for AFB1/T-2/ZEN, respectively. As long as the color contrast allows, it is feasible to utilize rainbow color tracers for the simultaneous detection of multiple analytes.

Cellulose nanoparticles coated with polydopamine were loaded with dyes to facilitate the competitive detection of multiple mycotoxins and to conduct a sandwich analysis of combined inflammatory biomarkers, thereby identifying the types of infections present. The colorimetric signal generated by these particles was notably more sensitive compared to AuNPs, owing to their larger size and brighter color, which enhanced the visual readout [71]. Visual labels greatly simplified the operation of LFT and results interpretation. The signal readout does not require additional instruments or equipment but only visual inspection. Thus, the assay cost is reduced in terms of manpower and time, and the efficiency is largely improved.

By employing a variety of labels including gold nanoparticles [57,72], colloidal carbon [66,68], and colored latex microspheres [73,74], LFT can achieve quick and straightforward result interpretation, making it suitable for a range of on-site test scenarios with limited resources [75]. However, despite the benefit of vibrant and high-contrast coloring, the human naked eye has its own visual limitations, which can impede the use of visual labels in the diagnosis of low-concentration analytes. In comparison to more recent technological advancements, the sensitivity and specificity of lateral flow tests based on latex microbeads may be moderately restricted. With ongoing progress and efforts, these markers will become more applicable for pathogen detection in LFT under more extensive and diverse conditions.

### 3.2. Markers Requiring Instrumental Analysis

The reliance on visual labels that can only be discerned by the naked eye significantly restricts the detection capabilities for analytes present in low concentrations. Therefore, there is a need for alternative labeling methods that can provide highly sensitive and precise test outcomes. There are several types of emerging markers used in LFT to indicate the captured analytes, including fluorescent labels, chemiluminescent labels and thermal contrast labels.

#### 3.2.1. Fluorescent Labels

Fluorescent markers require illumination with specific wavelengths of light, typically provided by a laser source, to produce fluorescence signals [76]. These signals can be observed with the naked eye or detected using specialized equipment like fluorescence microscopes [46] or other imaging tools [77,78] designed for signal analysis. Fluorescent labels present a viable solution to visible markers, as they exhibit a high signal-to-noise ratio. Additionally, fluorescent molecules are considerably smaller than colored beads, making them more comparable in size to the analytes in LFT. This size compatibility results in enhanced sensitivity of the signal, thereby enabling highly accurate quantitative assessments [79,80,81]. Various types of fluorescent labels, such as fluorescent dyes [76,82,83], fluorescent proteins [84], quantum dots [56,85,86], UCNPs [87,88,89], and polymer nanoparticles [90,91], have been implemented in LFT. Each label presents unique benefits and drawbacks. The careful selection of labels based on particular applications and experimental circumstances can significantly improve the sensitivity and accuracy of the tests.

Fluorescent readout in LFAs encompasses a diverse family of reporter molecules that share the common principle of photon emission upon excitation but differ fundamentally in their physical size, signal intensity per label, photostability, and compatibility with LFA membrane materials. It is essential to distinguish among these label types, as their properties dictate fundamentally different assay design considerations.

Label type classification. The major categories of fluorescent labels used in pathogen LFAs are as follows:

i. Free fluorescent dyes (e.g., fluorescein, Cy5, Alexa Fluor series). These are small organic molecules at the molecular scale (~0.5–1.5 nm) that are typically conjugated directly to detection antibodies. Their small size ensures rapid diffusion and minimal steric hindrance, but they carry only a single fluorophore per antibody, severely limiting per-binding-event signal output. Photobleaching is a significant concern, particularly under high-intensity excitation, and their signal is not visible to the naked eye, requiring a fluorescence reader. Free dyes have been largely superseded in LFA applications by higher-signal alternatives, though they remain relevant in some competitive-format LFAs for small-molecule targets.
ii. Dye-loaded polymeric or silica nanoparticles (e.g., Fluospheres^®^, europium-chelate-embedded polystyrene beads, dye-doped silica nanoparticles). These particles typically range from 20 nm to 500 nm in diameter—comparable to or larger than conventional 20–40 nm AuNPs—and encapsulate thousands to tens of thousands of fluorophores per particle. The high fluorophore payload provides substantial signal amplification per binding event compared to a single free dye molecule. However, the particle size must be carefully chosen: particles >200 nm may exhibit restricted mobility within the nitrocellulose membrane pore structure (typical pore sizes 3–20 μm for LFA membranes, but with effective constrictions that retard larger particles), potentially reducing binding efficiency at the test line and increasing nonspecific entrapment. Conversely, particles <50 nm carry fewer fluorophores but migrate more freely.
iii. Quantum dots (QDs) (e.g., CdSe/ZnS, InP/ZnS core–shell nanocrystals). QDs are semiconductor nanocrystals with typical core diameters of 2–10 nm, expanding to 15–30 nm after functionalization with a biocompatible shell and antibody conjugation. They offer exceptional brightness per particle, narrow and size-tunable emission spectra (enabling multiplexing), and resistance to photobleaching that far exceeds organic dyes. Their intermediate size places them between free dyes and bead-based labels. However, QDs are not inherently smaller than AuNPs: after full functionalization (core + shell + ligand layer + antibody), a QD conjugate may reach 25–35 nm, which is within the same size range as a 30–40 nm AuNP-antibody conjugate. Claims that QDs enable improved sensitivity because they are smaller are therefore not generally correct.
iv. Upconverting nanoparticles (UCNPs) (e.g., NaYF_4_:Yb,Er). UCNPs convert near-infrared excitation (typically 980 nm) to visible emission via an anti-Stokes mechanism, completely eliminating background autofluorescence from biological matrices and nitrocellulose membranes. Their sizes range from ~20 to 200 nm depending on synthesis conditions. Signal-to-background advantages—not size advantages—account for their reported sensitivity improvements.
v. Lanthanide chelate nanoparticles (e.g., Eu^3+^, Tb^3+^, Sm^3+^ chelate-loaded polystyrene or silica particles). These exploit the long luminescence lifetime of lanthanide ions (microseconds to milliseconds vs. nanoseconds for organic fluorophores) to enable time-resolved fluorescence detection, effectively gating out short-lifetime background fluorescence and scattered excitation light. Particle sizes typically range from 50 to 400 nm.

Size and sensitivity: a non-monotonic relationship. It is important to clarify that smaller label size does not inherently confer improved analytical sensitivity in LFAs. The relationship is governed by competing factors that act in opposite directions (Table 1).

Published experimental comparisons support the non-monotonic nature of this relationship. For example, in a systematic study of AuNP size in LFAs, nanoparticles of ~40 nm diameter provided optimal signal intensity in a sandwich-format LFA; smaller AuNPs (15 nm) produced weaker signal due to lower extinction per particle, while larger AuNPs (80–100 nm) showed reduced test line intensity attributed to restricted membrane mobility and steric effects. In the case of fluorescent labels, dye-loaded particles of 100–200 nm often outperform both smaller (<50 nm) and larger (>400 nm) particles for sandwich-format pathogen detection, as they balance adequate fluorophore payload (for strong signal) with acceptable membrane transport characteristics. Free fluorescent dyes, despite being the smallest possible label (~1 nm), are rarely employed in sandwich-format LFAs precisely because their single-fluorophore signal is insufficient for the required sensitivity.

In a study by Zhang et al. in 2022 [92], a lateral flow immunoassay chip using a second near-infrared (NIR-II) fluorescent dye encapsulated in polystyrene nanoparticles achieved a low detection limit for influenza A and influenza B viruses. This assay achieved a detection limit of 0.015 ng/mL for influenza A nucleoprotein and 10^2.08^ TCID_50_/mL (TCID_50_ refers to tissue culture infection dose at 50% endpoint) for influenza A virus, as well as 0.037 ng/mL and 10^0.43^ TCID_50_/mL for influenza B virus. In contrast, the visually inspected LFT with microbeads only achieves a LOD of 5.8 × 10^3^ plaque-forming units (PFU)/mL for influenza A and 8.2  ×  10^2^ PFU/mL (1 TCID50 ≈ 0.7 PFU) for influenza B virus, respectively [93].

**Figure 6:**
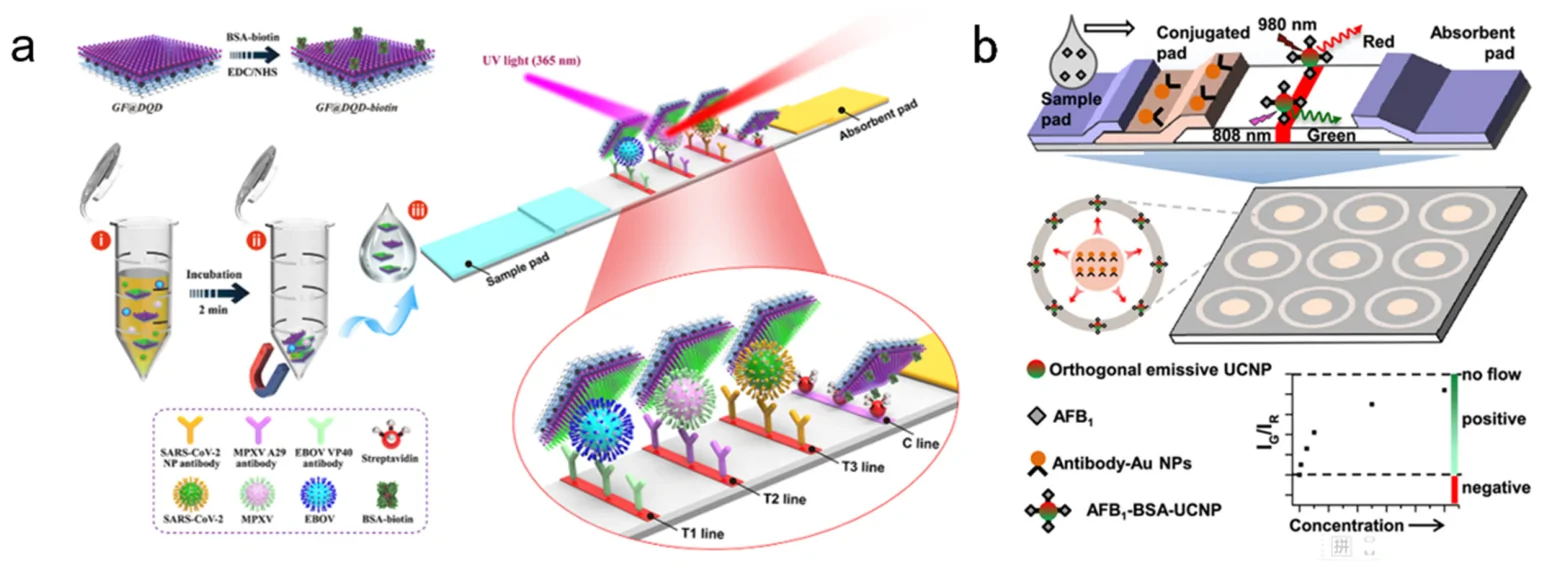
(**a**) Proposed LFIA biosensor for multiplex and ultrasensitive monitoring of EBOV, MPXV, and SARS-CoV-2 antigens. Reproduced with permission from Ref. [94]. Copyright 2024 ACS Nano. (**b**) Schematic Illustration of the Structure and Detection Principle of the Single-Line LFA Strip and Circular Flow Assay Strategy Based on the Single Test-Line Strips for multiple detections; the inset figure depicts the variations in intensity ratios (IG/IR) between the green emission on an 808 nm excitation and the red emission on a 980 nm excitation on the T line after samples with different amounts of analytes flowing across. Reproduced with permission from Ref. [96]. Copyright 2021 Analytical Chemistry.

Fluorescent inorganic nanomaterials, like quantum dots and UCNPs, offer advantages of high photostability and high fluorescence efficiency, making them suitable for broad-spectrum and ultra-sensitive virus/pathogen detection. Wang et al. [94] developed a multiplex LFIA platform that could achieve multiple virus tests on one strip with detection limits as low as pg/mL (Figure 6a). The design of the tracer for the LFIA contained two main parts: adsorption of Fe_3_O_4_ magnetic nanoparticles and fluorescent CdSe/ZnS-MPA quantum dots on a graphene oxide (GO) nanofilm (GF@DQD) as a versatile probe; conjugation of 4-aminophenylboronic acid (APBA) was used as a broad-spectrum recognition molecule. The test LODs for SARS-CoV-2 SP, MPXV A29, and EBOV GP antigens were 0.93, 1.03, and 0.89 pg/mL, which were 200 times lower than ELISA and 500 times lower than AuNP-based LFIA. The multiple tests in one strip could greatly reduce the assay time and cost. Hu et al. [95] developed highly luminous CdSe/ZnS quantum dots with hydrazide groups (QBs-NH-NH2) modified as the signal amplifier for LFA. The hydrazide-mediated oriented coupling facilitated antibody erection on the particle surface, thereby enhancing the bioactivity of the probes on luminescent nanomaterials. This strip exhibited a broad dynamic linearity, accommodating HBsAg concentrations ranging from 0.05 ng/mL to 3200 ng/mL with an LOD of 50 pg/mL.

Unlike quantum dots and other fluorescent molecules, upconversion nanoparticles (UCNPs) produce visible fluorescence when irradiated by NIR light. This distinctive characteristic allows UCNPs to potentially eliminate autofluorescence from biological samples and cross-talk fluorescence from other fluorophores, resulting in enhanced detection specificity. Additionally, the emission spectrum of UCNPs can be precisely adjusted within a specific range by incorporating selective doping ions. Moreover, it is feasible to engineer a single nanoparticle to emit two different colors of light under distinct excitation wavelengths. Leveraging this dual-emissive characteristic of UCNPs as a signaling mechanism, Guo et al. [96] have incorporated both test and control functionalities into a single line on an LFT strip (Figure 6b). The UCNPs are capable of emitting red light when excited at 980 nm and green light when excited at 800 nm, allowing one emission to act as a test signal and the other as a control or calibration signal. The test line has been innovatively designed in a circular configuration to enable multiple detection capabilities. By integrating several circular test lines onto a single strip, this design allows for high-throughput analysis, a feature that is not attainable with the conventional ladder-shaped strips. This represents a significant evolution from traditional LFT designs, enhancing diagnostic potential while also reducing the physical size and cost of the strips. With this innovative LFT, the LOD for aflatoxin B1 has been reported to reach as low as 25 ng/mL, demonstrating the effectiveness of this approach in sensitive detection.

Chen and colleagues [88] developed a dual-flux immuno-chromatographic assay (dICA) for the simultaneous quantification of two distinct mycotoxins, OTA and DON. In this study, upconversion nanoparticles (UCNPs) were utilized for fluorescence labeling of the antigen, while AuNPs acted as fluorescence quenchers to label monoclonal antibodies. The green fluorescence emitted by UCNPs at 540 nm was used as the analytical signal to indicate the formation of the antigen–antibody complex, confirming the presence or absence of the target analytes. Additionally, the red fluorescence emitted by UCNPs at 660 nm served as a quality control signal. By employing a dual-flux bidirectional single-line labeling mode, this assay enabled the simultaneous detection of two distinct mycotoxins on separate test lines. The study highlights the sensitivity, speed, and reliability of dICA for on-site detection of multiple mycotoxins.

**Figure 7:**
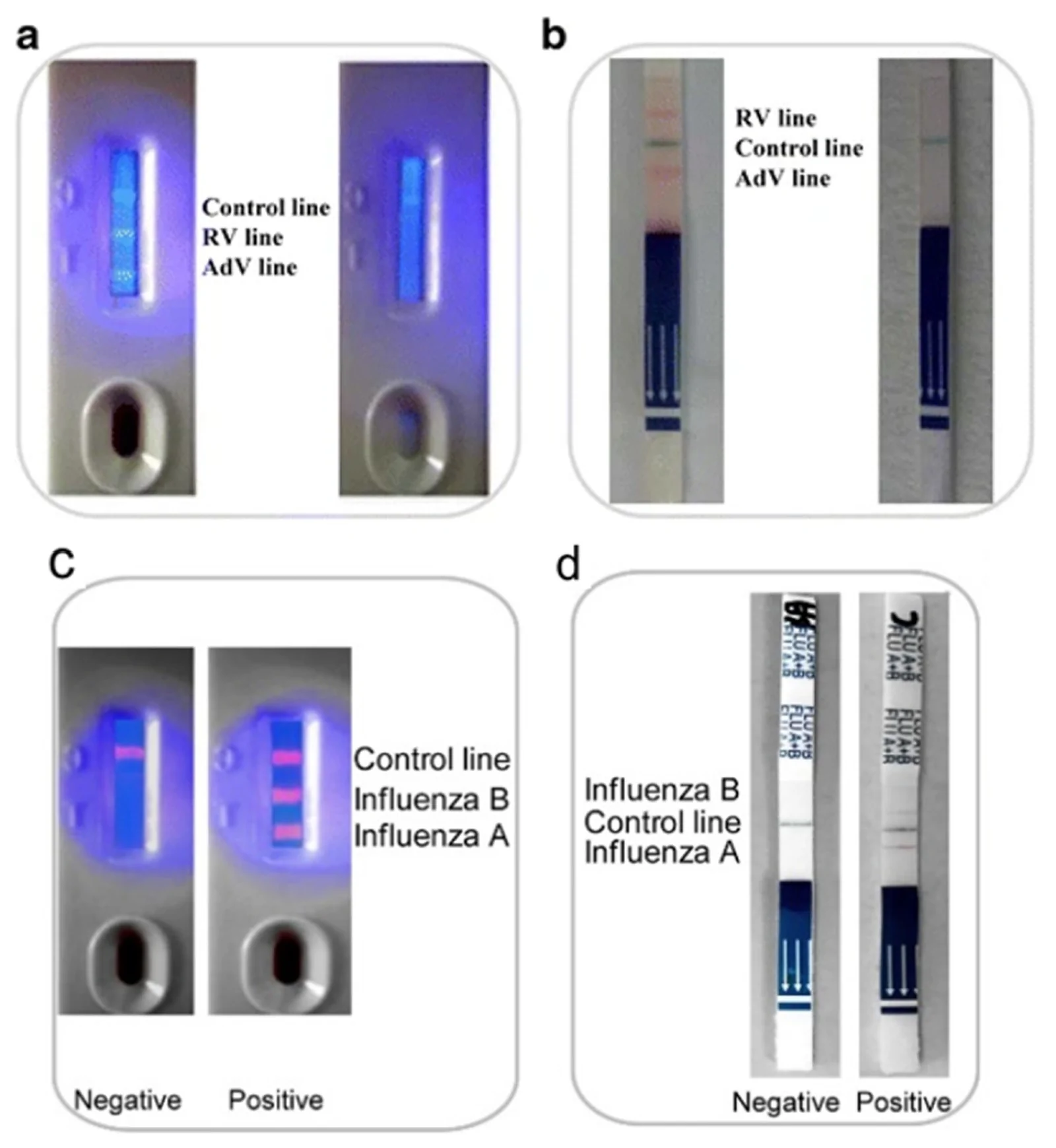
Comparison of performance for test strips with color beads and fluorescent beads as tracers. (**a**,**b**) Detection of rotavirus and adenovirus. Reprinted with permission from Ref. [69]. (**c**,**d**) Detection of influenza. Reproduced with permission from Ref. [93]. Copyright 2019 Virology Journal.

Jiang et al. [69] compared two commercially available test strips, one using colored microspheres as tracers while another using fluorescent microspheres (Figure 7a,b). The study focused on evaluating the performance and efficiency of these test strips for the detection of rotavirus and adenovirus in the stool specimens. Phenylethene luminative monomeric copolymers and blister were used as tracers in the two types of strips. Through analysis of 350 patients’ specimens, they found that sensitivity of fluorescent strips was significantly higher than that of color beads, but false positives occurred in the fluorescent test strips. The same research group [93] further compared two commercial test strips with fluorescent microspheres or colored microspheres as tracers for simultaneous detection of influenza A and B viruses (Figure 7c,d). Both test strips demonstrated excellent performance in terms of sensitivity and specificity, highlighting their potential for rapid influenza virus detection. In both of these research studies, the performance of fluorescent microsphere-based test strips surpassed that of colored microsphere-based test strips.

#### 3.2.2. Chemiluminescent Labels

Chemiluminescent molecules could emit light as a result of a chemical reaction. This distinct feature makes them exceptionally sensitive, ideal for the detection of even trace amounts of target molecules. When applied in lateral flow tests (LFTs), they offer a significant advantage over fluorescent labels by removing the need for external light sources, thus simplifying the detection process and enhancing its reliability. Typically, these labels comprise a substrate that interacts with an enzyme or catalyst to produce light. Frequently utilized enzymes in conjunction with these labels include horseradish peroxidase (HRP), alkaline phosphatase (AP), and luciferase. The enzyme facilitates the oxidation of the chemiluminescent substrate, resulting in the release of energy in the form of light. The intensity of the emitted light correlates directly with the quantity of the label present, enabling precise quantification of the target molecule. The signal generated at the molecular level facilitates improved precision in quantitative outcomes across various analytical and diagnostic assays.

In a lateral flow test, chemiluminescent molecules may serve as labels for antibodies or probes, producing light signals when they bind to the target pathogen. These signals are subsequently detected and quantified using photomultiplier tubes or charge-coupled devices (CCDs), which provide high sensitivity in measurement [97]. Han [98] reported a chemiluminescence (CL)-based LFA that involved a conjugation scheme using AuNPs, aldehyde-activated peroxidase and antibody to detect Cardiac Troponin I in human serum. HRP (horseradish peroxidase) catalyzed luminol-H_2_O_2_ chemiluminescence reaction facilitates quantitative detection, achieving a LOD of 5.6 pg·mL^−1^, which is a 110-fold increase in sensitivity compared to AuNP-Ab conjugate LFAs. g-C_3_N_4_/BiFeO_3_ nanocomposites were also utilized as chemiluminescent markers in LFIA for detection of endosulfan and imidacloprid, with both colorimetric and chemiluminescent signal dual-readout [99].

Chemiluminescent labels reduce interference and produce minimal background noise because the light emission is a result of a chemical reaction. These labels do not require external light sources, which is superior to fluorescent labels. Additionally, they could be used to perform quantitative analysis across a broad dynamic range. However, their use is limited by the need for rapid signal detection, as the emitted light decays quickly. Furthermore, the application of chemiluminescent labels involves more complex procedures compared to colorimetric or fluorescent labels, necessitating precise control over reaction conditions. These complexities have somewhat hindered their widespread adoption.

#### 3.2.3. Thermal Contrast Markers

In lateral flow assays, the binding of analytes to receptors can result in exothermic (heat-releasing) or endothermic (heat-absorbing) reactions, causing localized temperature changes. The thermal signals from specific and nonspecific bindings are distinct, enabling the sensitive and specific identification and quantification of the target analyte. Thermal Contrast Amplification (TCA) is an innovative method that enhances the sensitivity and detection capabilities of LFAs. It uses thermal imaging with a near-infrared (NIR) camera as an alternative to traditional colorimetric interpretation of LFA results. NIR camera can detect temperature variations as subtle as 0.1 °C, facilitating potentially quantitative analysis with a lower LOD. This approach harnesses the thermal responses to provide a sensitive and precise diagnostic tool.

For example, gold nanospheres (30 nm and 100 nm) and 150 nm gold nanoshells with silica cores were employed as TCA agents in the visual detection of HIV p24 protein [27]. The limits are reported to be 250, 62, and 62 pg/mL for 30 nm spheres, 100 nm spheres, and 150 nm shells, respectively. Furthermore, the thermal LOD for spiked p24 protein in human serum is determined to be 8 pg/mL, showcasing the potential of TCA in achieving highly sensitive and quantitative results in diagnostic applications.

TCA presents a compelling approach to boost the capabilities of lateral flow assays. However, it may necessitate the use of specialized temperature detection equipment. Thermal contrast materials are designed to exhibit property changes in response to temperature fluctuations, such as alterations in color, variations in emission intensity, shifts in wavelength, or modifications in temperature-dependent reflectivity and absorbance. These changes are correlated with the concentration of markers. Converting thermal detection into optical signals can be an effective alternative for enhancing the versatility and applicability of thermal-based assays. However, the temperature changes detected could be susceptible to interference from external temperature variations, potentially affecting the assay’s sensitivity.

Incorporating instrumentally analyzed labels in LFT significantly boosts the sensitivity and accuracy of pathogen detection. By employing fluorescent and chemiluminescent labels, LFT can provide rapid qualitative results and precise quantitative analysis. Application scenarios indeed impose specific requirements for diagnostic tests, such as the need for sensitivity and specificity when analyzing various sample types like blood, serum, or other body fluids. Additionally, the speed at which detection must be accomplished and the instrumentation available for use are critical factors. These requirements guide the selection and design of appropriate markers and detection methods to ensure test efficiency and reliability for each particular context. Ongoing advancements in labeling technologies are continually refining the precision, velocity, and ease of pathogen detection in LFT. These improvements are pivotal for more accurate disease diagnosis, effective monitoring, and enhanced control measures.

### 3.3. Physicochemical Determinants of Nanoreporter Performance

The performance of lateral flow assays is intrinsically linked to the physicochemical architecture of the nanoreporters. While increasing the hydrodynamic diameter of gold nanoparticles (AuNPs) generally enhances the Limit of Detection (LOD) due to increased optical density, it introduces a trade-off with transport kinetics; oversized particles may suffer from steric exclusion within the nitrocellulose matrix, reducing the effective capture probability. Furthermore, surface parameters—specifically Zeta potential—play a pivotal role in maintaining colloidal stability against the ionic strength of the sample matrix. An optimal nanoreporter must therefore balance high signal-per-particle (achieved via anisotropic morphologies like nanostars) with favorable hydrodynamic properties that ensure rapid, non-specific-binding-free migration through the porous membrane.

## 4. Applications of Lateral Flow Assays in Pathogen Diagnostics

### 4.1. LFAs Applied to Virus Detection

Viruses are ubiquitous in our environment, with their pathogenic nature leading to a variety of human diseases such as COVID, influenza, Ebola, HIV/AIDS, HBV, HPV [18,100,101,102,103], and others. The rapid replication and transmission of viruses have significant implications for public health, often resulting in fatalities and societal alarm [104]. Detecting virus infections is essential for individual isolation and for governments to implement effective control measures. LFAs offer a cost-effective and convenient method for early screening of infectious diseases.

#### 4.1.1. Detection of COVID

COVID, an acute infectious respiratory disease caused by the novel coronavirus SARS-CoV-2, emerged as a global pandemic in December 2019 [102]. Unlike previous outbreaks of severe acute respiratory syndrome (SARS) and Middle East respiratory syndrome (MERS) (outbreaks in 2002 and 2012, respectively [105]) caused by other coronaviruses, COVID has proven to be more widespread and transmissible. The number of infected people and the area affected by COVID overwhelmingly exceed those of SARS and MERS. One single COVID patient can infect more than three healthy people [106], as the virus primarily spreads through respiratory droplets. The clinical manifestations of COVID can range from mild to severe, with prevalent symptoms including fever, cough, and respiratory distress. Populations at heightened risk, particularly the elderly and those with pre-existing health conditions, are more susceptible to severe outcomes from the infection. Consequently, the creation of affordable and rapid diagnostic methods is essential for effectively managing the transmission of COVID.

The development of lateral flow tests (LFTs) for various infectious diseases typically requires several years; however, the antigen LFTs for SARS-CoV-2 were created and implemented within a few months. The Antigen Rapid Diagnostic Test was officially launched in the second quarter of 2021. The companies involved in the production of ART include Biomeme from the United States, Bioneer and SD Biosensor from South Korea, and Qlife from Sweden. By March 2022, more than 3 billion self-tests using LFA kits for SARS-CoV-2 had been conducted globally [107]. Given the rapid and extensive spread of the virus, the promotion of testing and immediate quarantine measures has become fundamental to our pandemic surveillance efforts, significantly aiding in the reduction of virus transmission.

**Figure 8:**
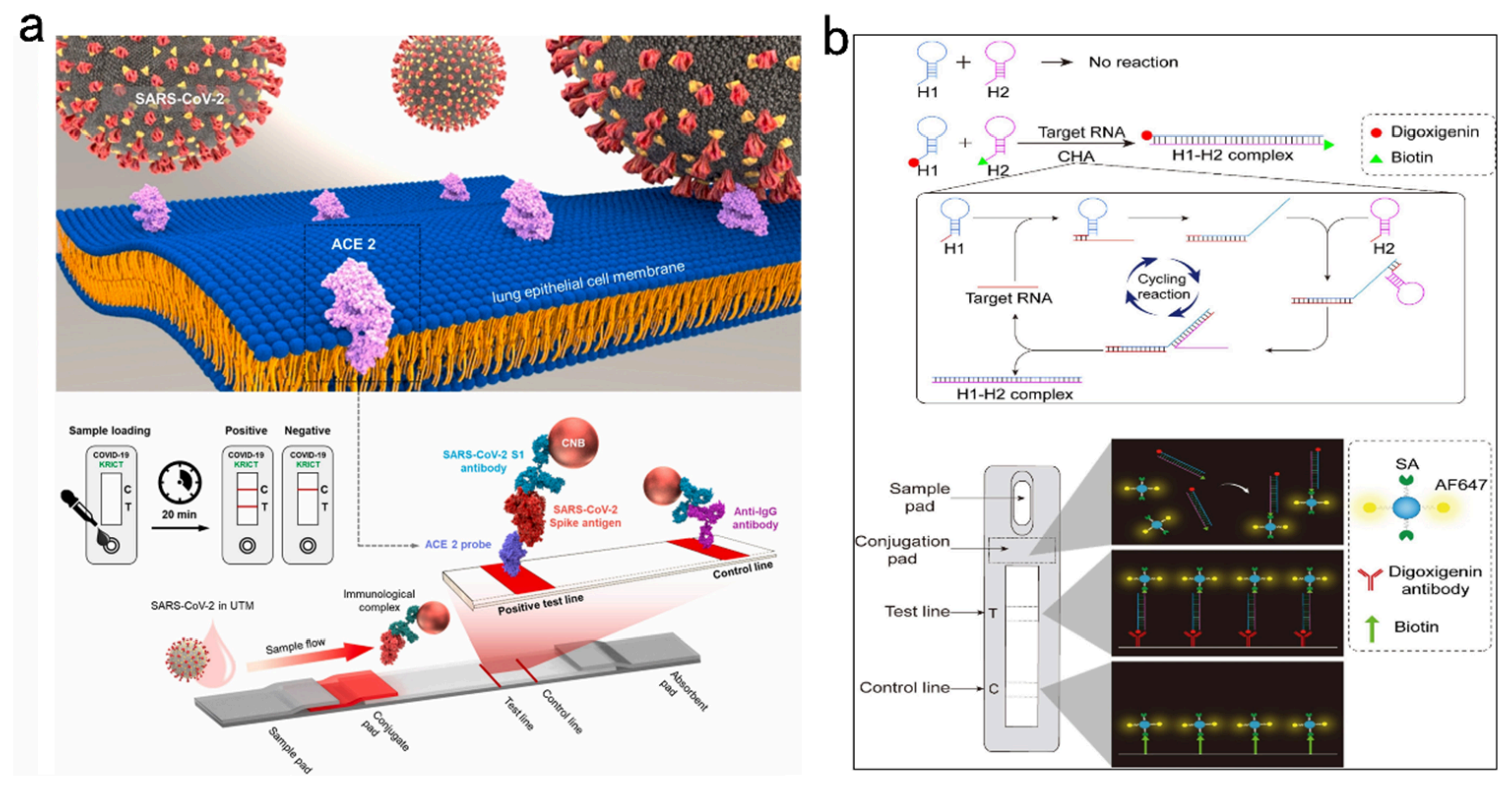
(**a**) Schematic diagram of SARS-CoV-2 recognition of ACE2 receptor and schematic diagram of ACE2-based composition and principle; the ACE2 receptor is positioned on the test line composed of a nitrocellulose membrane, while the control line is immobilized with an Anti-IgG antibody. Reproduced with permission from Ref. [110]. Copyright 2021 Biosens Bioelectron. (**b**) Schematic of the CHA-LFIA method for SARS-CoV-2 viral RNA detection, including the CHA reaction without and with the target RNA and a schematic diagram of the LFIA strip used for detecting digoxigenin-biotin labeled H1-H2 hybrid double-stranded complex. Reproduced with permission from Ref. [112]. Copyright 2021 Actuators B: Chemical.

Coronaviruses (CoVs) are enveloped, positive-sense RNA viruses. Within the SARS-CoV-2 virus, there are four major structural proteins: E (envelope protein), M (membrane protein), N (nucleocapsid protein), and S (spike protein) [108]. In the context of COVID detection, the S and N protein antigens are commonly used as biomarkers [109]. Kim et al. developed the first LFA [110] to identify SARS-CoV-2 spike S protein using commercial antibodies (S1-mAb) specific to angiotensin-converting enzyme 2 (ACE2), without cross-reactivity to MERS-CoV Spike 1 protein (Figure 8a). Red cellulose nanobeads conjugated with ACE2 were used as the detection probe. With a semi-quantified portable analyzer, the LOD was determined to be 1.86 ×10^5^ copies/mL, equivalent to less than 5 ng of recombinant protein in nasal-swab specimens. The detection process took 20 min. To increase specificity and sensitivity, a colorimetric and fluorescent bimodal LFA was developed for detection of SARS-CoV-2 S1 antibodies (subunit of the spike protein, with a LOD of 185 pg mL^−1^) and SARS-CoV-2 antigen (nucleocapsid (N)) protein (with a LOD of 212 pg mL^−1^) in nasopharyngeal swab samples [111]. In this study, polymer-coated gold nanorods were utilized as a versatile bimodal colorimetric and fluorescent reporter platform in LFAs. The colorimetric signals facilitated qualitative visual detection, enabling straightforward identification of targets through naked-eye observation. Complementarily, the fluorescence signals permitted precise quantitative analysis, particularly at low concentration limits, thereby enhancing the sensitivity and reliability of the detection method.

Besides protein, nucleic acid is also used. Zou et al. [112] developed an LFA strip for detecting SARS-CoV-2 RNA sequences in oropharyngeal swab samples without RNA isolation (Figure 8b). The assay involves the conditional catalysis of two single-stranded hairpin DNA molecules, H1 and H2, by the target RNA sequence. The presence of the target RNA is confirmed by the detection of H1-H2 hybrid duplexes. Initially, the RNA is amplified through a catalytic hairpin assembly (CHA) reaction. Streptavidin-conjugated Polyethylene (PE) nanoparticles labeled with the fluorophore Alexa Fluor 647 are immobilized on the conjugation pad. Mouse anti-digoxin/digoxigenin monoclonal antibodies were fixed on the test line, and biotin on the control line. In this format, recombinant H1-H2 hemagglutinin is dual-labeled with digoxigenin (DIG) and Alexa Fluor 647. The DIG tag mediates capture at the test line via high-affinity binding to immobilized anti-digoxigenin antibody, while the Alexa Fluor 647 conjugate provides the fluorescence readout. Thus, the fluorescence signal intensity at the test line is proportional to the amount of DIG–H1-H2–AF647 captured. The LOD for SARS-CoV-2 RNA was determined to be 2000 copies/mL.

**Figure 9:**
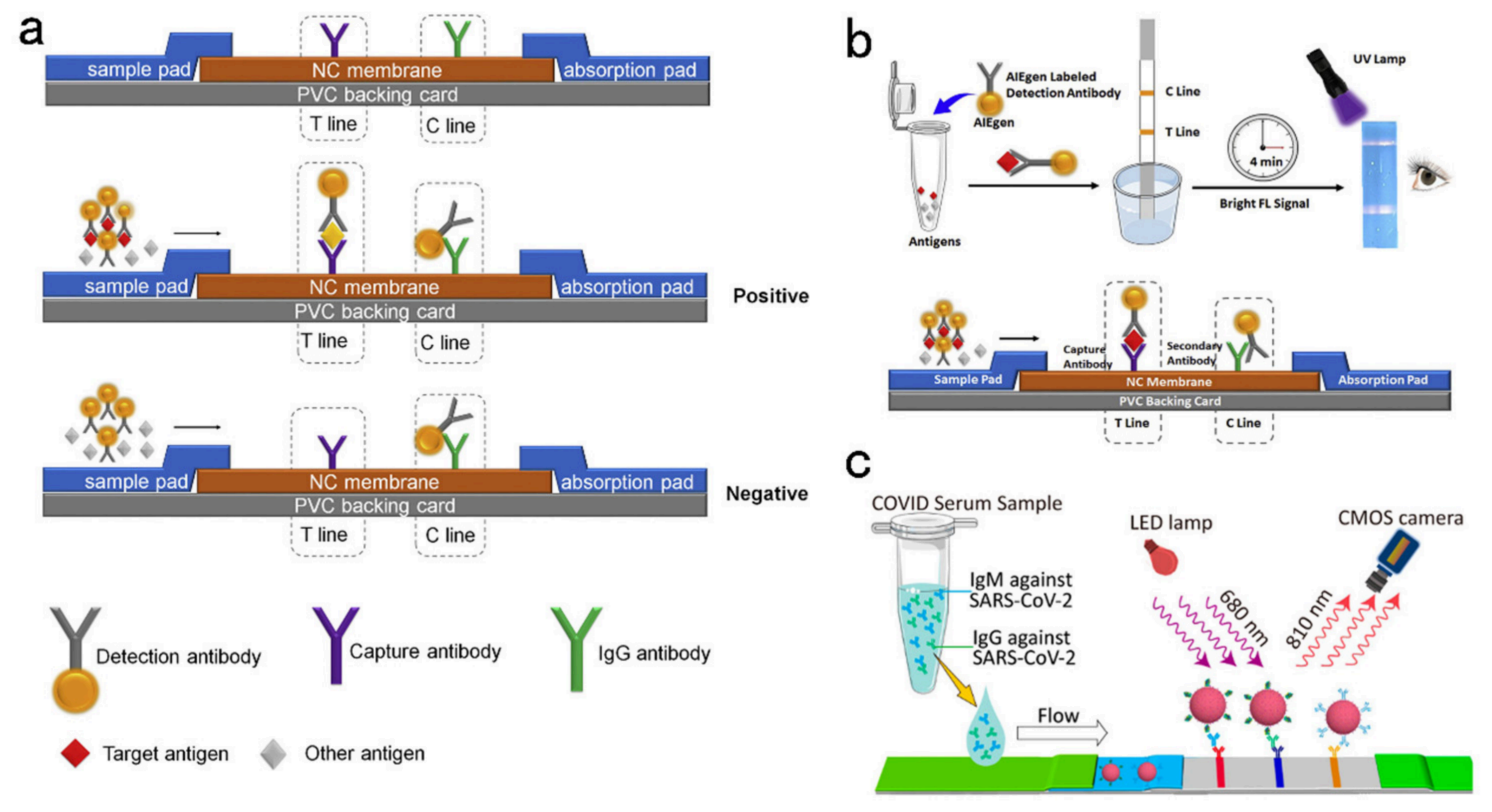
(**a**) Schematic of the configuration and the detection mechanism of AIEgen-based test strips. (**b**) Schematic diagram of an AIEgen-based wearable lateral flow strip for the rapid detection of SARS-CoV-2 RBD protein and N protein. Reproduced with permission from Ref. [114]. Copyright 2022 Cell Reports Physical Science. (**c**) Schematic illustration of test strips for the detection of the NIR-emissive AIE nanoparticle-labeled lateral flow immunoassay for detection of IgM and IgG in human serum samples. Reproduced with permission from Ref. [115]. Copyright 2021 ACS Nano.

Viral RNAs exhibit lower stability compared to proteins like antigens and antibodies [113], making them more suitable for transport and storage. Zhang and colleagues introduced a sandwich test strip for COVID antigen detection utilizing Aggregation-Induced Emission (AIE) luminogens as a reporter [114]. The test line trapped the binary complexes of antigen and AIE-labeled antibody, and the aggregation-induced fluorescence signal was then read upon illumination of a 365 nm UV lamp. This assay demonstrated a LOD of 5.5 ng/mL for C-reactive protein (CRP), 6.9 ng/mL for the receptor binding domain (RBD) protein, and 7.2 ng/mL for the N protein (Figure 9a,b). Another study utilized AIE nanoparticles as the fluorescent marker for the early detection of COVID-19-specific antibodies, immunoglobulin M (IgM) and immunoglobulin G (IgG) against SARS-CoV-2 in human serum samples (Figure 9c) [115]. The AIE nanoparticles exhibit fluorescence in the NIR region, effectively reducing background and noise from autofluorescence. The detection limits for IgM and IgG were determined to be 0.236 and 0.125 μg mL^−1^, respectively. Following the analysis of 172 serum samples, the test strip exhibited a sensitivity of 78% for IgM and 95% for IgG, surpassing the performance of ELISA and colloidal AuNP-based test strips. The sensitivity levels achieved by this lateral immunoassay were comparable to those of ELISA and superior to the colloidal AuNP-based test strip.

#### 4.1.2. Detection of Influenza Virus

Influenza virus can disseminate rapidly through the air or by contact with contaminated surfaces, thus emerging as a significant public health issue. Symptoms exhibited by patients often mirror those of COVID, including fever, cough, sore throat, body aches, and fatigue. The emergence of a drug-resistant pandemic H1N1 (pH1N1) virus in 2009 triggered a global outbreak that claimed the lives of around 20,000 individuals [116]. Early detection and adherence to preventive measures are key in managing the spread of influenza and preventing serious complications.

Influenza infections are caused by negative-strand RNA viruses, including Influenza A viruses (IAVs), Influenza B viruses (IBVs) and Influenza C viruses (ICVs) [117]. There are two types of influenza globally: sporadic pandemics caused by IAVs and epidemic (seasonal or interepidemic) influenza caused by IAVs and IBVs [118]. IAVs are zoonotic pathogens that can spread and mutate in various animal hosts, such as birds, pigs, and humans. IAVs are classified based on the presence of two viral surface proteins, hemagglutinin (HA) and neuraminidase (NA), with16 HA and 9 NA subtypes. Unlike IBV, IAV has the capacity to cause a pandemic by undergoing reassortment between avian and human IAVs [8]. Among the IAV subtypes, H1N1 and H3N2 are the most common subtypes causing disease in humans.

**Figure 10:**
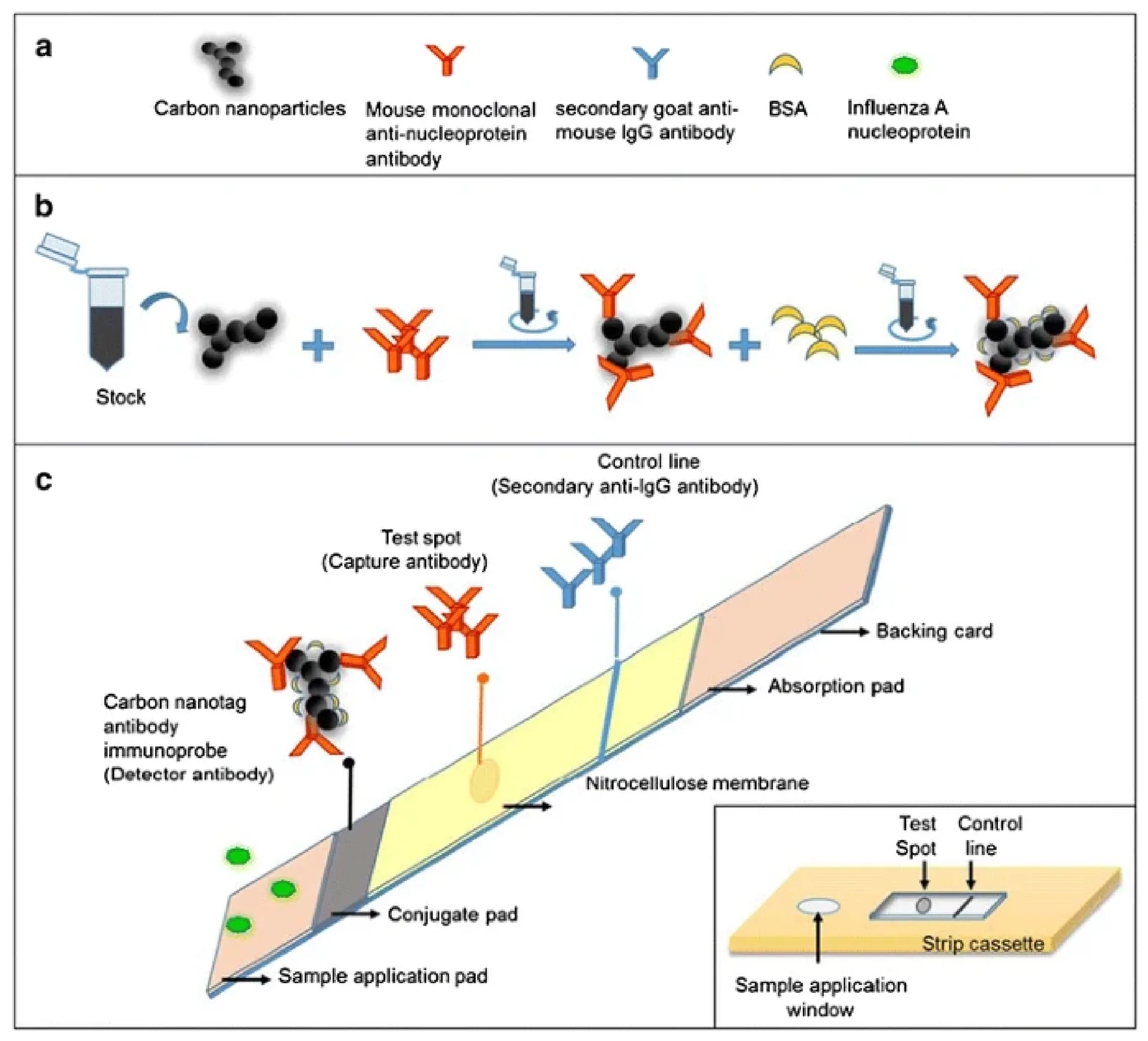
Schematic illustration of LFA design in the diagnosis of influenza A virus strains H1N1 and H3N2. (**a**) An overview of the components and (**b**) procedures for preparation of the carbon nanotag-antibody immunoprobes, and (**c**) the fabrication of carbon nanotag-based lateral flow immunoassay. Reproduced with permission from Ref. [119]. Copyright 2017 Microchimica Acta.

In 2017, Wiriyachaiporn et al. reported an LFA for the detection of influenza A virus H1N1 and H3N2 strains from cell lysates using an antibody–antigen-carbon nanotag antibody complex [119], achieving a LOD of 350 TCID_50_.mL^−1^ (Figure 10). Carbon nanoparticles in the form of nanostrings were employed as signal reporters in the LFA, allowing for the direct visualization of the analytical signal.

The researchers observed that the incorporation of SERS tags led to a significant improvement in the LOD, reducing it by nearly 40 times compared to traditional visual detection methods. Wang et al. [120] conducted a study on a quantitative LFIA using SERS for the detection of influenza A H1N1 virus and human adenovirus (HAdV), where Fe_3_O_4_@Ag nanoparticles were utilized as magnetic SERS nanotags. The test results were visible to the naked eye at concentrations of 10^5^ PFU/mL for H1N1 and 10^4^ PFU/mL for HAdV. In 2024, Zhu et al. [121] developed gold-shell silica-core nanoparticles for use as a SERS substrate, which was subsequently integrated into a lateral flow test strip for the purpose of detecting IAV. In conjunction with enzyme Recombinase Polymerase Amplification (RPA), the LFT demonstrated an impressive sensitivity of 10^5^ copies/mL for the DNA standard of the IAV matrix gene. This enhancement in sensitivity allowed for the quantitative analysis of the virus within a linear range spanning from 2.63 × 10^3^ copies/mL to 10^9^ copies/mL, providing a reliable and accurate means of virus detection and quantification. Other methods, such as an entropy-driven hairpin-free amplification approach, have also been integrated into LFT for H1N1 detection [122]. This entropy-driven mechanism facilitates isothermal nucleic acid amplification. With DNA coupled to fluorescent nanospheres (FNs) as detection markers, the lateral flow assay exhibited high sensitivity in detecting H1N1-RNA, achieving a remarkably low detection limit of 2.02 pM.

Combining an aptamer and an antibody in a single lateral flow assay has also been explored to enhance the test specificity. Le et al. [123] demonstrated this by directly detecting whole influenza H3N2 virus particles, achieving a detection limit of 2 × 10^6^ virus particles. This approach integrates the specificity of aptamers with the rapid binding kinetics of streptavidin-biotin to address issues like indistinct control lines.

Due to the similarity in symptoms, research has been conducted to differentiate SARS-CoV-2 infection from influenza. For instance, Lee et al. [124] reported a multiplex lateral flow detection for detecting SARS-CoV-2, as well as influenza A and B viruses in human saliva. The assay features a colorimetric signal amplification process to improve sensitivity. This method demonstrated a remarkable ability to detect SARS-CoV-2, influenza A, and influenza B viruses, achieving a sensitivity that is approximately 25 times higher compared to traditional commercial lateral flow assays.

#### 4.1.3. Detection of Hepatitis

Hepatitis is primarily caused by viruses such as hepatitis B and C, which invade the body via bloodborne infections, mainly damaging the liver. Chronic hepatitis B infection poses a significant risk of severe liver damage and an increased likelihood of developing liver cancer. Early detection is crucial for prompt treatment and preventing transmission. The primary diagnostic method for hepatitis B and C involves blood tests for antibodies, followed by confirmatory tests to assess the viral load in the liver and bloodstream. The presence of hepatitis B surface antigen (HBsAg) in the serum is a key diagnostic marker for HBV infection [125]. For example, Liang et al. [126] developed a competitive LFA for the quantitative detection of hepatitis B core antigens, which used.

**Figure 11:**
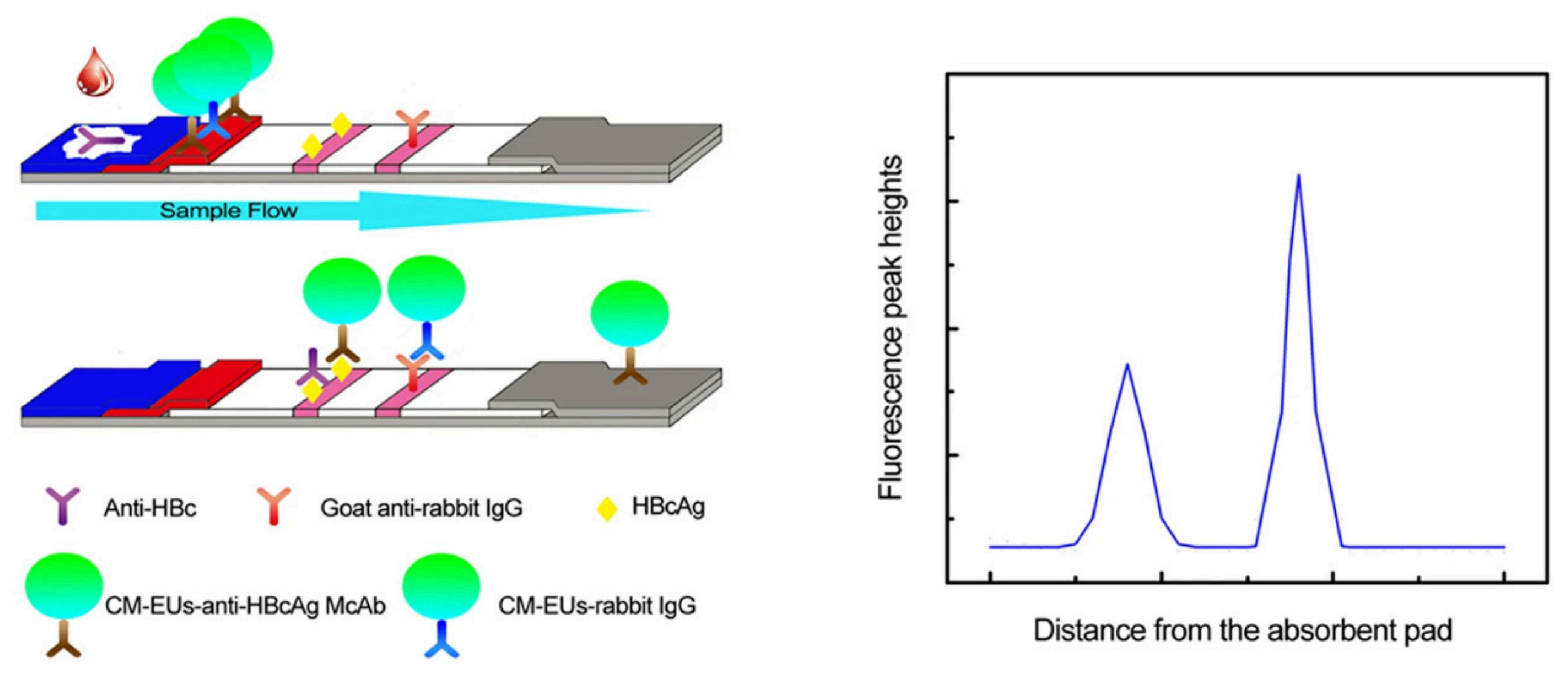
Schematic depiction of the CM-EUs-based lateral flow assay. The recombinant HBcAg is immobilized on the test line, whereas the anti-RIgG is positioned on the control line. In the assay, the anti-HBcAg monoclonal antibody competes with the anti-HBc present in the sample to bind with the immobilized protein. Quantitative detection is achieved by analyzing the fluorescence signal recorded using a portable TRF strip reader (Darui, Guangzhou, China), enabling accurate and rapid results. Reproduced with permission from Ref. [126].

Eu (III) chelate microparticles as a reporter (Figure 11). The assay displayed a LOD of 0.31 IU/mL and a linear range from 0.63 to 640 IU/mL for the quantification of anti-hepatitis B core antigen (anti-HBc) levels.

**Figure 12:**
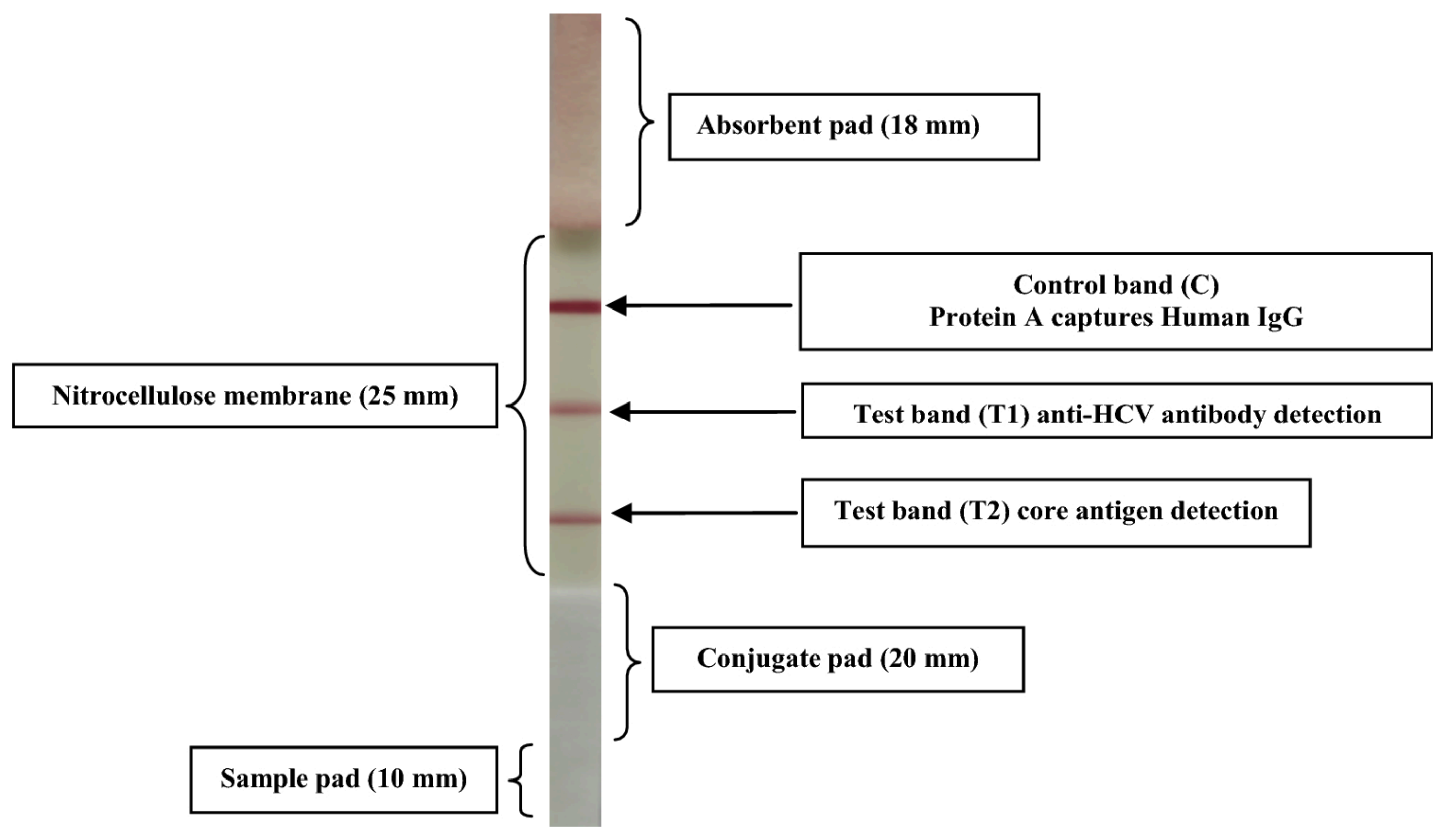
Schematic showing the HCV Ag-Ab Combo test strip. Reproduced with permission from Ref. [131].

In contrast to HBV, Hepatitis C virus (HCV) is an enveloped single-stranded, positive-sense RNA virus belonging to the family Flaviviridae [127]. This family also includes the yellow fever virus, dengue virus, West Nile virus, and Zika virus [128]. The HCV protein system consists of non-structural proteins (NS2, NS3, NS4A, NS4B, NS5A and NS5B) as well as structural proteins (core, E1, E2 and p7) [129]. It is widely acknowledged that core antigen can be identified within 1 to 2 days following HCV RNA detection, typically during the pre-seroconversion stage. For RNA detection, amplification techniques, particularly LAMP (Loop-Mediated Isothermal Amplification), have usually been integrated into LFA to enhance the test’s sensitivity. Therefore, antigen detection could serve as an alternative to HCV RNA detection in resource-limited settings [130]. However, HCV core antigen becomes undetectable when anti-HCV antibodies are present in the blood. Patel et al. [131] addressed the challenge with an antigen–antibody combination-based LFA, enabling the simultaneous detection of HCV core antigen and anti-HCV antibodies (Figure 12). The assay is capable of identifying HCV infection approximately 7 to 12 days earlier than traditional anti-HCV antibody detection methods, thereby significantly enhancing the early diagnosis of HCV. Notably, the assay demonstrated no false-negative results when tested with 27 samples that were strongly positive for anti-HCV antibodies, as well as serum samples that were spiked with recombinant HCV core antigen at concentrations ranging from 1.37 to 1000 ng/mL.

Notably, the commercialization of LFA for HBV detection has made significant progress, with companies such as Abbott leading the way. The diagnostic sensitivity and specificity of HBsAg (Hepatitis B Surface Antigen) LFAs have achieved remarkable levels, exceeding 99% and 98%, respectively, thereby ensuring highly accurate results.

#### 4.1.4. Detection of HIV

The human immunodeficiency virus type 1 (HIV-1) specifically targets white blood cells, leading to a progressive deterioration of the body’s immune system. This infection renders individuals more vulnerable to a myriad of diseases and infections, with symptoms ranging from flu-like illness in the early stages to the development of Acquired Immunodeficiency Syndrome (AIDS) in the most advanced stage [132]. Patients infected with HIV typically produce antibodies against the virus as part of the immune response, usually within a 28-day window following infection. The timely and precise diagnosis of HIV is of paramount importance in preventing the spread of infections, enabling the commencement of appropriate treatment, and significantly reducing mortality rates linked to the virus. Key viral markers such as anti-HIV antibodies [133], HIV-1 capsid protein (p24 antigen) [134,135] and HIV-1 RNA [136] are commonly utilized in the detection of HIV infection.

The primary methods for detecting HIV involve identifying antibodies in either blood or saliva samples, where techniques such as enzyme immunoassays (EIAs) and Western blot analysis are typically employed to ascertain the presence of the virus. It is essential to stress that following potential high-risk exposure to HIV, a confirmatory test should be performed by healthcare professionals, especially if the initial test results are negative. This is due to the possibility of low antibody levels during the window period, which could lead to an inaccurate result. Additionally, rapid antibody tests are insufficient for diagnosing HIV in infants under 18 months old. In such cases, nucleic acid tests (NATs) are recommended to directly identify the viral genetic material in the blood, ensuring a more accurate diagnosis.

Deng and colleagues created LFA strips that utilize CdTe-H2 quantum dots to detect HIV-DNA [11]. The assay was able to achieve a LOD as low as 0.76 pM (S/N = 3) and a detection range spanning from 1 pM to 10 nM. Choo et al. [137] improved the quantitative detection of HIV-1 DNA by incorporating SERS into LFA. As an exceptional optical-enhancing agent in surface-enhanced Raman spectroscopy (SERS), AuNPs significantly augment the SERS signals of the Raman reporter molecule, 4-(dimethylamino)azobenzene-4′-thiol (MGITC). This enhancement effect remarkably boosts the detection sensitivity, enabling more precise and efficient analysis. This study successfully achieved a remarkable detection limit of 0.24 pg/mL. In comparison with a commercially available fluorescence assay kit designed for HIV-1 DNA detection, the minimum detectable concentration in this study was determined to be 80 pg/mL.

Integrating a test strip with a mobile device not only holds the potential to significantly reduce errors in interpretation and transcription but also has the potential to minimize the need for comprehensive workforce training, thereby enhancing efficiency and streamlining processes. In a study conducted by I. Martiskainene et al. [138], the double-antigen bridge assay format LFA was created for the identification of anti-HIV-1 and HIV-2 antibodies, where upconversion nanoparticles (UCNPs) were employed as reporters [139]. The test outcomes could be conveniently accessed through a mobile phone application, and the platform facilitates the seamless integration and transfer of data to electronic health record systems, enhancing the overall management and accessibility of patient information. This integration not only streamlines clinical workflows but also contributes to more informed decision-making in healthcare settings.

#### 4.1.5. Detection of HPV

**Figure 13:**
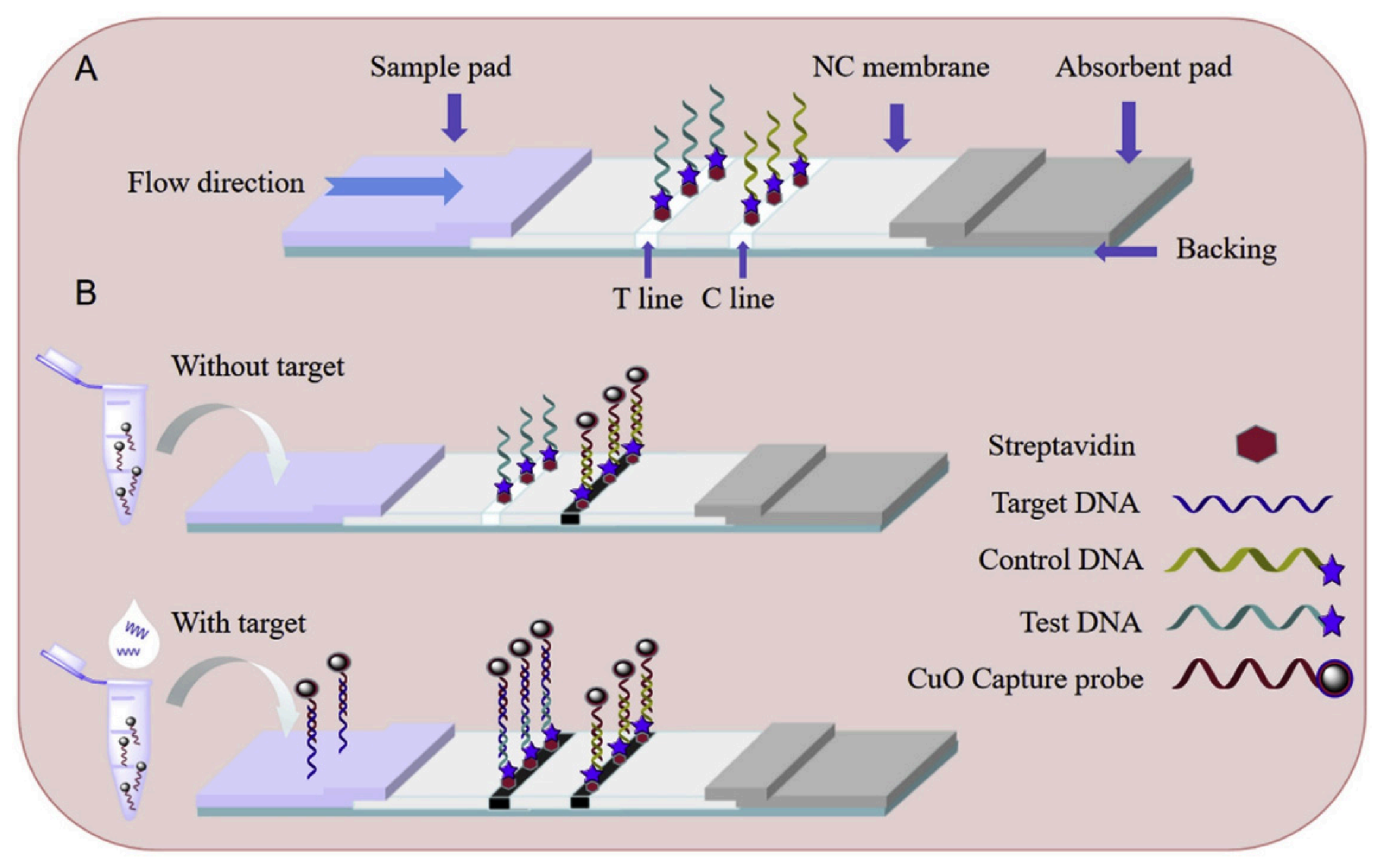
Schematic illustration of the CuO-based LFT to detect HPV16 DNA. (**A**) Structural composition of the lateral flow strip. (**B**) Detection mechanism of the assay. Top: In the absence of target DNA, the CuO capture probes do not bind to the T line, resulting in no signal at the test line. Bottom: In the presence of target DNA, a sandwich complex is formed on the T line via hybridization with the test DNA and control DNA, allowing the accumulation of CuO capture probes and generating a visible signal. The legend indicates Streptavidin, Target DNA, Control DNA, Test DNA, and the CuO Capture probe. Reproduced with permission from Ref. [142].

Human papillomavirus (HPV) is a non-enveloped, double-stranded DNA virus that has been implicated in over 5% of all cancer cases worldwide. These include various forms of cancer such as head and neck cancer, oropharynx cancer, vaginal cancer, penile cancer, anus cancer, and most significantly, cervical cancer [140]. Of the 15 high-risk oncogenic HPV genotypes identified, HPV16 and HPV18 are responsible for approximately 70% of cervical cancer cases. Consequently, current strategies for early detection and intervention primarily concentrate on these two distinct HPV types. For instance, Chen et al. [141] developed an LFA capable of simultaneously detecting HPV16 and HPV18 in clinical specimens. The AuNP-FITC antibody conjugate was employed to bind the target DNA, thereby facilitating the transmission of colorimetric signals. In conjunction with PCR, this biosensor demonstrated the capability to detect HPV16 and HPV18 DNA with remarkable sensitivity, with a minimum concentration threshold of 700 copies. The PCR can complicate detection and delay results. In a study using CuO NPs as markers, PCR or other amplification procedures were omitted, yet HPV16 was still quantitatively detected (Figure 13) [142]. The LFA allowed visual qualitative detection and quantitative analysis through measuring the peak area of the test line on the Kinbio strip reader. The CuO-based LFA has a detection range of 5 nM to 100 nM, with an LOD of 1.0 nM.

#### 4.1.6. Detection of Ebola Virus

On 15 May 2026, the Democratic Republic of the Congo (DRC) and Uganda jointly announced a new outbreak of Ebola, marking the 17th occurrence of the disease in the Congo since its initial discovery in 1976. As of 18 Jul 2026, official reports from DRC indicate a total of 2344 cases confirmed through laboratory testing, and tragically, 930 fatalities [143].

The Ebola virus is an extremely transmissible and frequently fatal virus that leads to Ebola virus disease (EVD) in humans and other primates. The virus spreads primarily through contact with the bodily fluids of infected individuals or animals. Due to its rapid transmission and high fatality rate among humans, Ebola virus disease poses a substantial health threat for affected areas, particularly in West Africa. This alarming mortality rate significantly complicated and impeded ongoing research endeavors. Tragically, a genomic sequencing study resulted in the deaths of six authors prior to its publication in the journal Science in 2014 [144]. In response to the urgent need for diagnostic tools, the World Health Organization (WHO) granted emergency authorization in March 2015 for the first lateral flow assay (LFA) for EVD, designating it as a valuable supplementary resource to enhance PCR testing capabilities.

**Figure 14:**
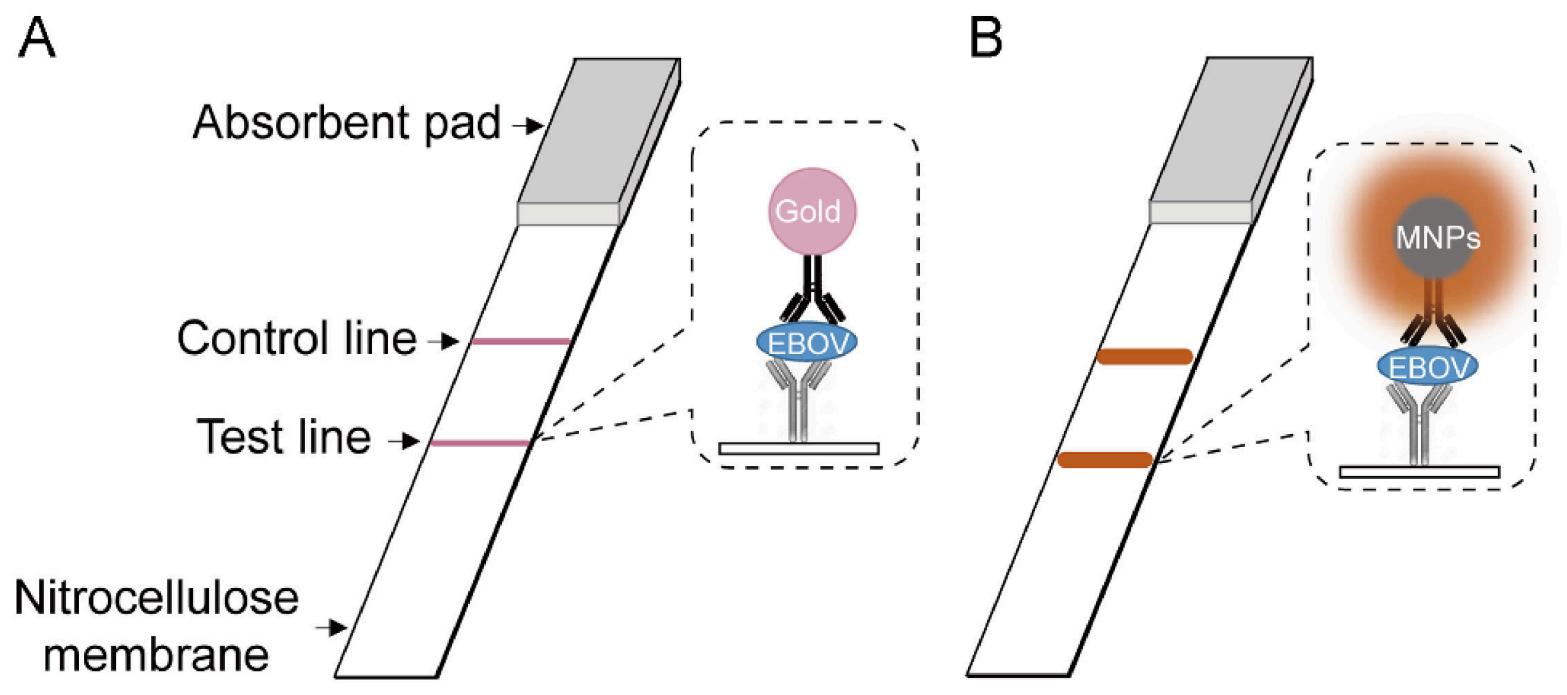
(**A**) Standard colloidal gold strip. (**B**) Nanozyme-strip employing MNPs in place of colloidal gold to form a novel nanozyme probe. The probe with nanozyme activity generates a color reaction with substrates, which significantly enhances the signal so that it can be visualized by the naked eye. Nanozyme-strip design. Reproduced with permission from [145].

Magnetic nanoparticles (MNPs) are capable of catalyzing peroxidase substrates and leading to an obvious color reaction, reminiscent of peroxidase-like activity, thus earning them the classification as nanozymes. Drawing on this property, Duan and colleagues created an LFA strip with magnetic nanoparticles (MNPs) of Fe_3_O_4_ as a reporter to detect the glycoprotein (GP) of the Ebola virus (EBOV-GP) (Figure 14). This approach demonstrates a sensitivity 100 times greater than that of gold nanoparticles (Au NPs), enabling the detection of EBOV-GP at an impressively low concentration of 1 ng/mL through visual observation alone [145].

**Figure 15:**
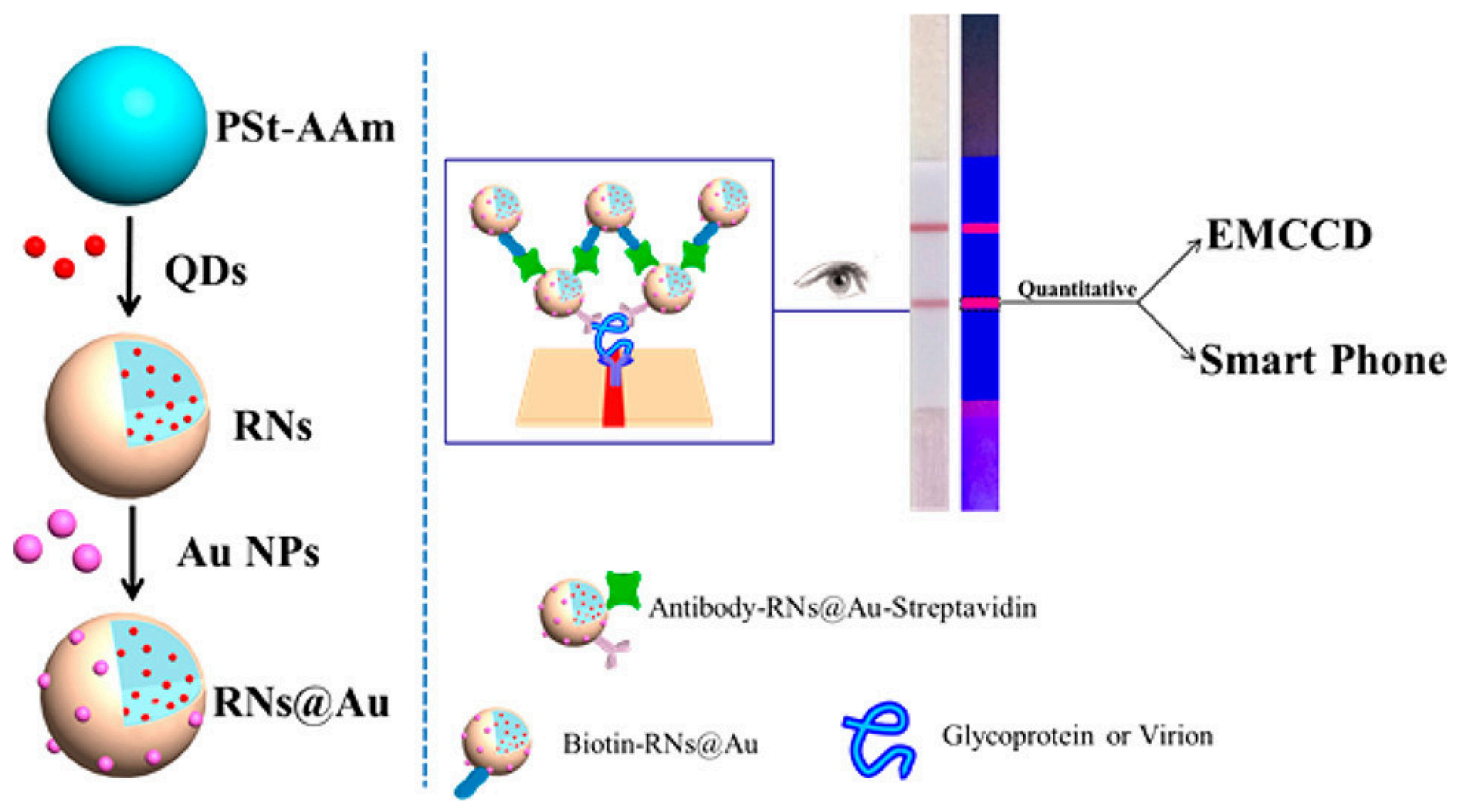
Design of Lateral Flow Immunoassay for the detection of Ebola with RNs@Au-based nanospheres. Reprint permission obtained from [146].

A nanosphere composed of CdSe/ZnS QD and AuNP was also used as the reporting mechanism in LFA for detection of Ebola virus (EBOV) glycoprotein [146]. This reporter integrates hundreds of quantum dots and dozens of AuNPs (RNs@Au) into a poly(styrene/acrylamide) copolymer nanosphere, enabling a dual-signal readout that combines fluorescence for quantitative analysis with a colorimetric signal for visual identification (Figure 15). The analyte capture is achieved through the interaction of biotin-avidin pairs, with antibodies and streptavidins conjugated to the RNs@Au. The lateral flow assay demonstrates the capability of naked-eye detection of glycoprotein at a concentration of 2 ng/mL within a 20 min timeframe, while the quantitative detection limit was established at 0.18 ng/mL, showcasing its high sensitivity and efficiency.

**Figure 16:**
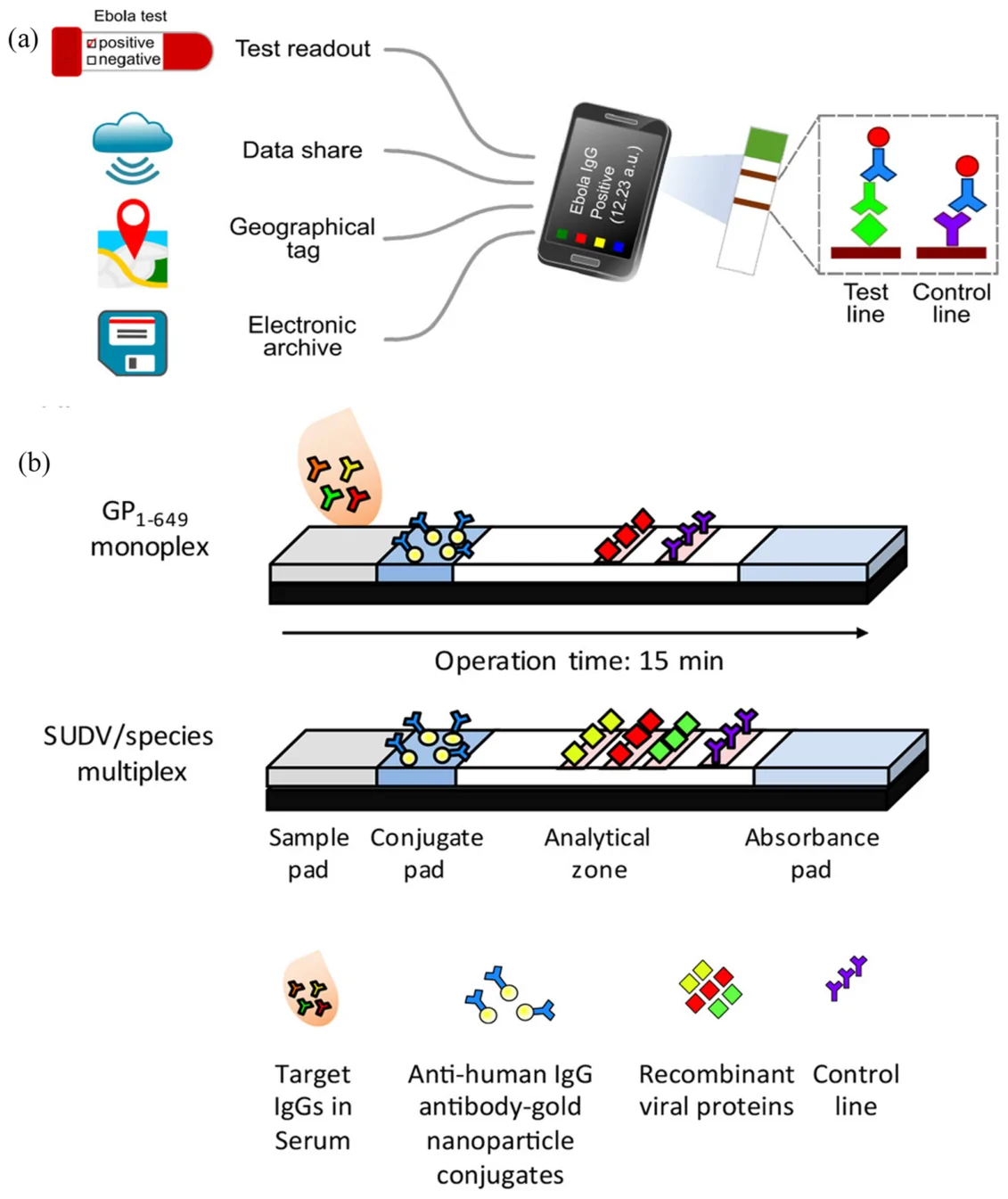
(**a**) Illustration of a smartphone lateral flow point-of-care test for Ebola virus IgG detection. (**b**) Lateral flow strip illustration: serum applied onto the sample pad migrates through the analytical area, and subsequently forms complexes between the labeled gold nanoparticles (AuNPs) and the target analytes. Reprint permission obtained from [147].

Recovery from EVD in human survivors triggers a rapid development of both cell-mediated and humoral immune responses. In light of this, Polina et al. developed a Serological LFIA (Figure 16) for the Detection of IgG Antibodies against Ebola Virus in Human Survivors [147]. This innovative diagnostic tool utilizes the body’s immune response to detect the presence of IgG antibodies specifically targeting the Ebola virus. In conjunction with a smartphone reader, it is capable of detecting and semiquantifying Ebola-specific antibodies in survivors. The performance of the strip validated a remarkable 100% sensitivity and 98% specificity with freshly collected patient samples in Uganda.

Given the rapid transmission and high mortality rate associated with the Ebola virus, the swift screening and timely diagnosis made possible by lateral flow tests (LFTs) become especially invaluable in the context of an outbreak. In a race against time to save lives, the potential limitations of LFTs are often outweighed by their utility in facilitating effective patient management and implementing containment strategies.

#### 4.1.7. Detection of Dengue Virus

Dengue, a debilitating infectious disease spread by mosquitoes, is caused by one of four serotypes of the dengue virus (DENV1-DENV4) [148]. The disease predominates in tropical and subtropical climates, and its symptoms closely resemble those of influenza [149]. The clinical manifestations of dengue include a characteristic fever, persistent headaches, intense muscle and joint pain, as well as skin rashes. Treatment for dengue does not involve a specific therapeutic regimen; instead, it emphasizes supportive care aimed at pain relief and the restoration of fluids and electrolytes. Furthermore, there are currently no fully approved vaccines for dengue, and prevention relies solely on vector control measures to reduce the likelihood of transmission.

Dengue IgM antibodies are a reliable marker of recent infection but not necessarily acute infection. The quantity of nonstructural protein 1 (NS1) antigen released has been recognized as a potential marker for assessing the severity of the disease [150]. Panbio, Biorad, and SD are among the companies that have developed LFA strips to detect dengue virus NS1 antigen and IgM, IgG, and IgA antibodies. These strips have been in use since the 1990s and have advanced to the third generation [151]. These LFAs consist of nitrocellulose strips with colloidal gold-labeled monoclonal antibodies serving as reporters, exhibiting consistent specificity ranging from 92.5 to 100%.

The aggregation of AuNPs on the conjugate release pad often leads to elevated background signals, thereby reducing sensitivity. To address this issue, Chen and his colleagues [7] developed a lateral flow assay (LFA) strip that employed the near-infrared (NIR) fluorescent dye DyLight-800, enhancing the detection of anti-DENV1 IgG antibodies. Through the analysis of 19 primary clinical samples, they discovered that the sensitivity of the NIR-LFA significantly exceeded that of the Panbio IgG ELISA and the Dengue Duo IgM/IgG Cassette, achieving a remarkable twofold improvement in the lower limit of detection. This innovation holds great promise for more accurate and efficient diagnostic applications.

Additionally, the use of different labeling techniques, such as TEMPO, Au-rGO, and FND, demonstrates the versatility and potential of lateral flow assays in improving diagnostic capabilities. Sinawang et al. [152] developed an LFA with hydrophilic redox label TEMPO, capable of detecting dengue NS1 protein at concentrations as low as 50 ng/mL. Another study by Kumar et al. [153] introduced a lateral flow assay for the detection of dengue NS1, employing gold-decorated graphene oxide (Au-rGO) sheets as labels. This innovative method achieved a remarkable detection limit of 4.9 ng/mL, showcasing its efficacy in identifying dengue NS1. Le et al. [154] developed an LFIA using Fluorescent Nanodiamonds (FND) as reporting agents. The negatively charged nitrogen-vacancy centers in FND can alter the electromagnetic field, triggering fluorescence. This fluorescence is used as the reporter in LFA, allowing for the detection of NS1 serotypes (DV1, DV2, DV3, and DV4) with a limit of 0.1 to 1.3 ng/mL. RT-RPA was also employed for rapid DENV detection in lateral flow strips [155]. These advancements in diagnostic techniques have significant implications for the early detection and management of dengue fever. The ability to detect the virus at such low concentrations can lead to faster and more accurate diagnoses, ultimately reducing the spread of the disease.

#### 4.1.8. Detection of Zika Virus

Zika virus (ZIKV) is primarily spread by Aedes mosquitoes and can also be transmitted sexually and from mother to fetus during pregnancy. Since its identification in 1947, Zika virus has emerged as a significant global public health concern [156]. The 2015 outbreak in Brazil led to 4000 cases of microcephaly and 1.5 million infections. Many infected individuals are asymptomatic, but symptoms can include fever, rash, joint pain, and conjunctivitis. Zika infection in pregnancy is associated with severe birth defects like microcephaly. There is no specific treatment, so management focuses on supportive care and minimizing mosquito exposure.

Similar to other members of the Flaviviruses genus, ZIKV is an enveloped virus characterized by a single-stranded positive-polarity RNA. The conventional analysis of Zika virus (ZIKV) relied on the detection of specific DNA sequences following reverse transcription-polymerase chain reaction (RT-PCR). However, recent research has delved into the application of the loop-mediated isothermal amplification (LAMP) platform for the detection of amplicons, offering a potentially more efficient and rapid diagnostic approach. Lee et al. [157] utilized LFA to identify the amplicons of RNA products following treatment of the sample with Bst 3.0 polymerase and optimized Reverse Transcription Loop-Mediated Isothermal Amplification (RT-LAMP). This amplification technique specifically targets Zika virus (ZIKV) RNA in pure water and human whole blood, thereby eliminating the need for exogenous reverse transcriptase. In a similar vein, Gna Ahn et al. [158] conducted a study utilizing LFA to identify RT-LAMP amplicons of ZIKV DNA, employing the fluorescent indicator SYBR Green I. They utilized six primers designed to target specific regions on the DNA strands for detection. The specificity of the ZIKV RT-LAMP test in human samples reached 100% at a concentration of 1 × 10^3^ PFU.

**Figure 17:**
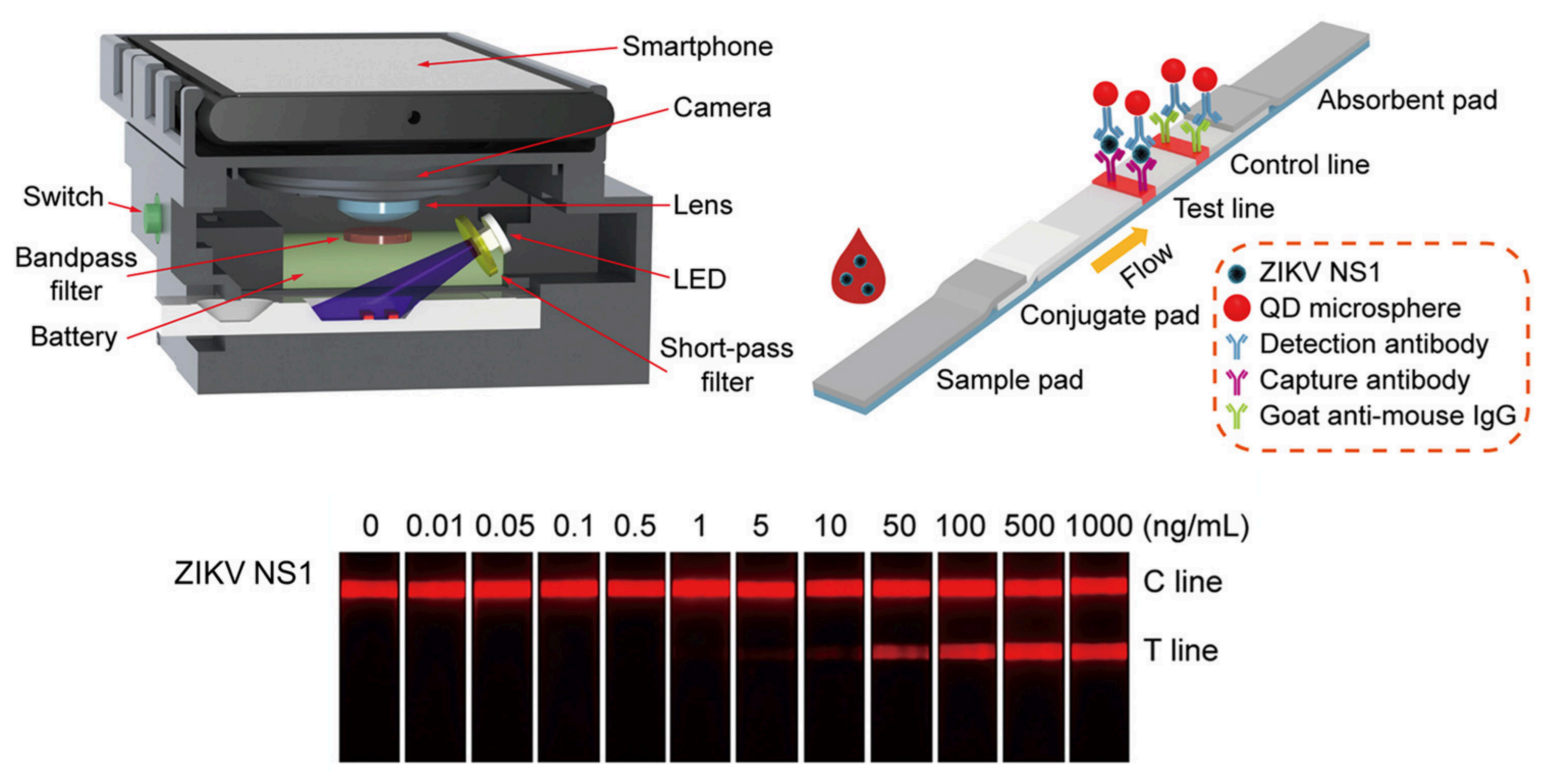
Portable smartphone-based fluorescent LFIA reader for highly sensitive point-of-care detection of ZIKV NS1. Reprint permission from [165].

In addition to diagnosing nucleic acid amplicons, LFA is also designed to identify specific proteins associated with ZIKV. ZIKV RNA genome encodes a single polyprotein. This polyprotein is associated with multiple copies of the capsid protein, all encapsulated within an icosahedral structure [159]. ZIKV consists of three structural proteins-capsid, and seven nonstructural proteins, namely NS1, NS2A, NS2B, NS3, NS4A, NS4B and NS5 [160,161]. Among these, nonstructural protein 1 (NS1) is secreted by cells infected with the virus and interacts with the host [162]. Within the cellular environment, NS1 forms homodimers and associates with the adipocyte membrane system, which plays a role in viral replication [163]. As a key antigen, NS1 can induce the production of antibodies, making it a valuable target for the early diagnosis of viral infections [164]. Nevertheless, the antigenic resemblance between Zika virus (ZIKV) and dengue virus leads to serological cross-reactivity, which hinders the ability of antibody-based tests to accurately differentiate between infections caused by ZIKV and dengue virus. Rong. et al. [165] introduced an LFA platform for identification of the Zika virus nonstructural protein 1 (ZIKV NS1) with CdSe/ZnS quantum dot microspheres as fluorescent probes (Figure 17). The intensity of the test line of ZIKV NS1 demonstrated a sigmoidal relationship within a concentration range of 0.01 to 1000 ng/mL, achieving LODs of 0.045 ng/mL in buffer and 0.15 ng/mL in serum. This system incorporated external optical and electrical elements into a smartphone readout platform to enhance user convenience. Li. et al. [166] created four monoclonal antibodies (mAbs), designated A1, B1, C1, and 9E-1, that specifically target the envelope protein of ZIKV. Among these, mAbs B1 and 9E-1 were selected for incorporation into an LFIA aimed at detecting the ZIKV envelope protein and virions, utilizing AuNPs as the reporter in a traditional LFA design.

A comprehensive summary of recent studies focusing on the detection of viral pathogens using various LFA platforms is presented in Table 2.

### 4.2. Detection of Bacteria

Bacteria are unicellular prokaryotic organisms that are ubiquitous on Earth. They are known for their simple cellular structure, which does not include a nucleus or membrane-bound organelles, distinguishing them from eukaryotic cells. Bacteria exhibit an extraordinary degree of diversity, and they can be found in nearly every habitat, from deep-sea vents to the Arctic ice, and even reside within living organisms. Bacteria’s impact on our world is profound, with both beneficial and detrimental effects. Their influence spans various aspects of life, with both positive and negative outcomes. For example, bacteria play pivotal roles in ecosystems by breaking down organic matter and facilitating nutrient cycles. However, excessive bacterial growth, such as the pollution in the Rein River that delayed Olympic events, can have adverse effects. Meanwhile, the beneficial bacteria residing in our colons are essential for our health and bodily functions. Conversely, certain bacteria can cause serious illnesses, including tuberculosis, streptococcal infections, and Lyme disease, or lead to foodborne poisoning. The detection of bacteria is crucial for protecting our health. For accurate identification, tests may assess bacterial morphology, antibiotic resistance, and genetic material. Given the microscopic size and the fact that sophisticated detection equipment may not always be accessible, LFTs provide a convenient and rapid alternative for preliminary bacterial detection. A brief overview is presented in Table 3.

#### 4.2.1. Detection of *Treponema pallidum*

*Treponema pallidum* is a bacterium causing syphilis, a sexually transmitted infection that can also be passed from an infected mother to her child during gestation. Within the genus Treponema, four subspecies are known to infect humans: *T. pallidum* subsp. *pallidum*, *T. pallidum* subsp. *pertenue* (responsible for yaws), *T. pallidum* subsp. *endemicum* (associated with bejel or endemic syphilis), and *T. carateum* (which causes pinta) [171]. All four pathogenic spirochetes exhibit similar morphological characteristics and can be identified through serological tests that detect antibodies for syphilis.

**Figure 18:**
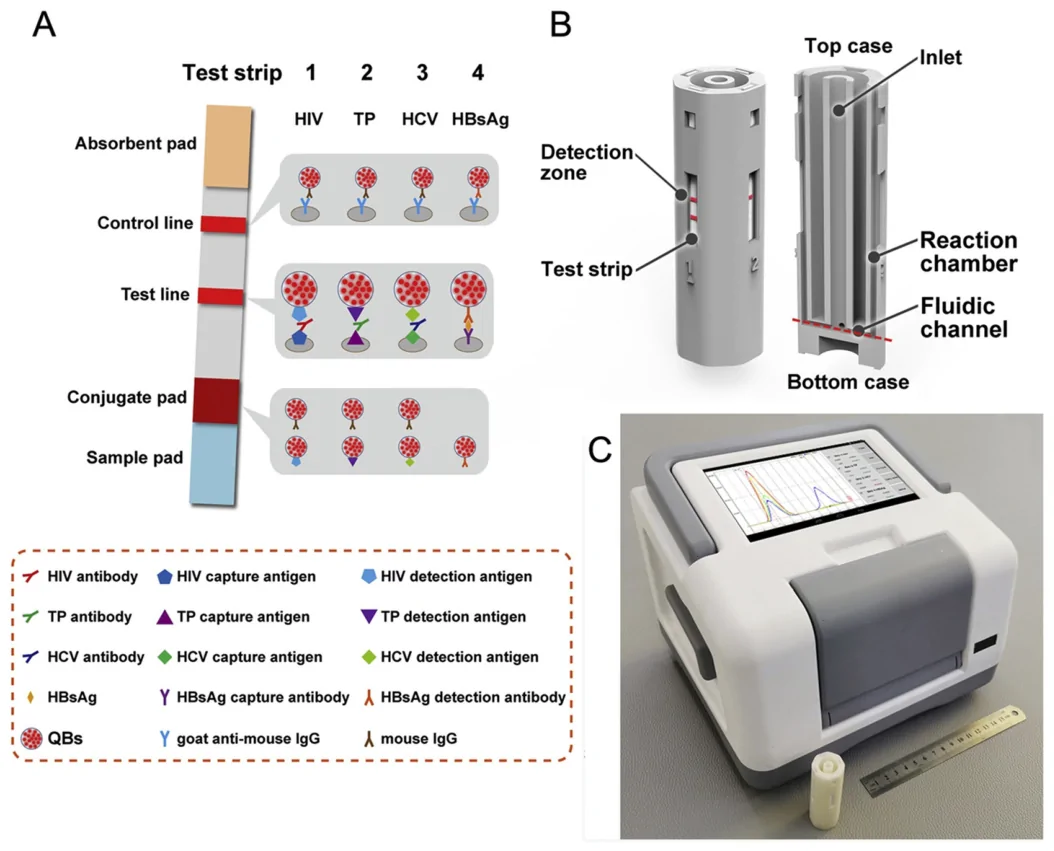
Schematic illustration of the integrated lateral flow assay platform. (**A**) Illustration of integrated fluorescent lateral flow assay for multiplex detection of infectious diseases (HIV, TP, HCV, and Hepatitis B Virus). (**B**) Design of disposable strip test cartridge. (**C**) Image of portable strip reader and test cartridge. Reproduced with permission from Ref. [173]. Copyright 2021 Sensors and Actuators B: Chemical.

Among these pathogens, *T. pallidum* is regarded as the most virulent due to its unique ability to breach both the blood–brain barrier and the maternal-fetal barrier. Recent studies have indicated that polyelectrolyte-coated gold magnetic nanoparticles (PGMNs) can facilitate the rapid and accurate detection of antibodies against *Treponema pallidum* [172]. Rong et al. developed a comprehensive LFA platform capable of simultaneous multiplex detection for four biomarkers of infectious diseases from clinical serum samples [173]. These targets include three human antibodies specific to HIV, syphilis (TP), and HCV, as well as one antigen, HBsAg (Figure 18). Utilizing quantum dot beads as markers, the assay demonstrated low limits of detection: 0.11 NCU/mL for the HIV antibody, 0.62 IU/L for the TP antibody, 0.14 NCU/mL for the HCV antibody, and 0.22 IU/mL for HBsAg. The entire assay process was completed within a 20 min timeframe.

#### 4.2.2. Detection of *Escherichia coli* O157: H7

*Escherichia coli*, a Gram-negative bacillus, is a common resident in the lower intestines of warm-blooded animals, where it typically exists symbiotically. The majority of *E. coli* strains are benign and even beneficial to human digestion. However, certain strains, such as O157:H7, produce Shiga toxins, which can lead to serious foodborne diseases [174]. These include gastrointestinal illness, Hemolytic Uremic Syndrome (HUS), and Thrombotic Thrombocytopenic Purpura (TTP). The ‘H7’ in its name indicates the specific flagellar antigen, which is associated with the bacterium’s mobility. The strain *E. coli* O157:H7 poses a significant threat to public health due to its propensity to trigger severe diseases and its regular involvement in foodborne illness outbreaks. Therefore, prompt and precise detection of *E. coli* O157:H7 is essential in preventing widespread disease.

Ye and colleagues created a combined test strip designed for the specific detection of *E. coli* O157:H7 by concurrently identifying both the O157 antigen and the H7 antigen [175]. The assay utilized monoclonal antibodies that were generated using classical hybridoma technology by the research team, achieving a detection sensitivity of 1 × 10^4 colony-forming units (CFU) per milliliter. Research by Shi et al. [176] demonstrated that a SERS-based LFIA could significantly enhance the sensitivity for identifying *E. coli* O157:H7 by 2000-fold, achieving a LOD as low as 50 cells per milliliter in a PBS sample. In this study, gold-shell silica-core (SiO_2_/Au) nanospheres served as SERS tags to enhance the system’s sensitivity.

In another advancement in 2021, Hasan and colleagues [177] developed a method that combines magnetic enrichment of bacteria from real samples with rapid SERS detection, enabling selective isolation and enrichment of *E. coli*. The researchers used the rennet enzyme to detach casein-modified Fe_3_O_4_/Au-PEI nanoparticles from the primary antibody-bound bacteria, preventing nanoparticle aggregation. They established a linear correlation between the concentration of *E. coli* and the SERS signal in the range of 10^1^ to 10^7^ CFU/mL (with a coefficient of determination, R^2^, of 0.984), and they determined the LOD to be 0.52 CFU/mL.

A label-free LFA has been developed for rapid detection of *E. coli* O157: H7, eliminating the need for paired antibodies and traditional labeled probes in strip-based sensors [178]. In this assay, nanozyme-mannose-modified Prussian blue (man-PB) nanoparticles serve dual roles as both the recognition agent and the signal indicator. The man-PB nanoparticles facilitate three signal readout formats in the strip: qualitative detection through naked-eye readout, quantitative detection by analyzing the signal intensity on T line, and the colorimetric signal after catalytic reaction. This LFA demonstrates superior performance in detecting target pathogens, with a detection range spanning from 10^2 to^ 10^8^ CFU·mL^−1^ and a low limit of detection at 10^2^ CFU/mL, adaptable to various readout methods.

Alternative labeling techniques have been utilized in the detection of *E. coli*. For instance, UCNPs functioned as reporters in a sandwich LFIA for identifying *E. coli* O157:H7 [179]. The use of Cucurbit [7] uril-modified UCNPs has shown potential to enhance sensitivity and reduce costs. Additionally, 2,3-Bis(4-(bis(4-(tert-butyl)phenyl)amino)phenyl)fumaronitrile (BAPF), an aggregation-induced emission luminogen, has been employed for both qualitative and quantitative analysis of *E. coli* [180], offering a sensitive and cost-effective detection approach.

#### 4.2.3. Detection of *Listeria monocytogenes*

*Listeria monocytogenes* is a rod-shaped, Gram-positive bacterium that can cause the serious foodborne illness known as listeriosis. This infection has the potential to escalate into severe health issues, including meningitis, sepsis, and adverse pregnancy outcomes such as miscarriages or stillbirths [181]. This bacterium is particularly noteworthy for its ability to multiply and establish biofilms at temperatures below 0 °C, a condition commonly found in household refrigerators. Moreover, it exhibits resistance to various disinfectants and has the potential to contaminate a wide array of food products, such as unpasteurized dairy products, fresh fruits and vegetables, and various types of meat [182]. Therefore, the identification of *L. monocytogenes* is crucial to mitigate the risk of infection.

**Figure 19:**
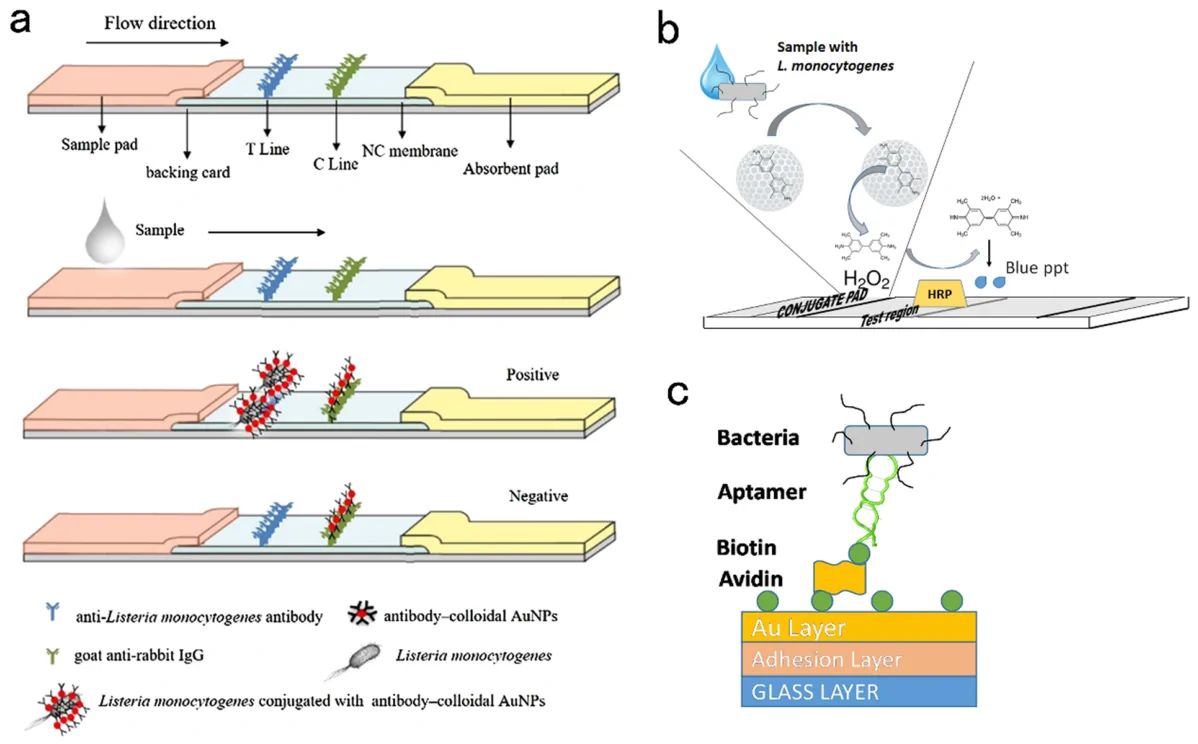
(**a**) Schematic of LFIA strip structure and detection principle. Reproduced with permission from Ref. [183]. Copyright 2020 Journal of Materials Science. (**b**) Schematic representation of assay principle. *L. monocytogenes* cells migrate to the conjugation pad region, where binding to the aptamer is gated. (**c**) Schematic representation of the aptamer-gated label-free LFA. Reproduced with permission from Ref. [185]. Copyright 2019 Analytical Biochemistry.

Du et al. developed an LFIA for *L. monocytogenes* detection, using AuNPs as a labeling agent [183]. The assay employed 60 μg/mL of L. monocytogenes antibody, achieving detection limits of 2.5 × 10^5^ CFU/mL in pure cultures of L. monocytogenes and 2.85 × 10^5^ CFU/mL in samples of pork tenderloin (Figure 19a). The incorporation of SERS technique markedly improves the sensitivity of LFIA. The enhancement could result in detection limits as low as 75 cfu/mL, with linear responses observed within the concentration ranges of 10^2^–10^7^ cfu/mL with for *L. monocytogenes* [184] (Figure 19b).

Aptamer-gated LFA also demonstrated notable specificity in the detection of L. monocytogenes. B. Busra described a label-free Aptamer-gated LFA, which employs the release of 3, 3′, 5, 5′-Tetramethylbenzidine (TMB) as a reporter molecule [185]. The release of TMB occurs upon bio-affinity interactions, subsequently reacting with horseradish peroxidase immobilized in the test region, resulting in the formation of blue particles through enzymatic conversion (Figure 19c). The identification of pretreated bacteria was facilitated by biotin-avidin specific binding. Avidin-coated gold slides served as the substrate of test region. One end of the aptamer, which was conjugated to biotin, was utilized to capture the target bacteria, while the opposite end of the aptamer sequence included an additional six nucleotides at the 3′ terminus to specifically bind to L. monocytogenes. The assay achieved a LOD of 53 cells/mL within a timeframe of less than five minutes. Additional detection details and supplementary information, categorized by their respective reporter molecules, are summarized in Table 4.

## 5. From Bench to Field: Clinical Translation and Commercialization of Pathogen LFAs

### 5.1. Overview of Commercially Available Pathogen LFAs

While the preceding sections of this review have focused on emerging research-stage LFA formats with novel reporter molecules and signal amplification strategies, it is important to recognize that a mature commercial ecosystem of pathogen LFAs already exists. The global lateral flow assay development services market reached an estimated USD 3.36 billion in 2025 and is projected to grow to USD 5.4 billion by 2032, reflecting sustained demand for point-of-need testing across food safety, clinical diagnostics, veterinary, and environmental monitoring sectors. Within food safety specifically, commercially available LFA products for pathogen detection have been marketed for over two decades by both multinational corporations and specialized diagnostic companies.

Representative commercial products include (Table 5).

A key observation from this commercial landscape is that nearly all commercially validated LFAs for foodborne pathogens retain a culture-based enrichment step (typically 12–48 h) prior to LFA testing. This reflects a fundamental trade-off in practical pathogen detection: while research-stage LFAs increasingly report enrichment-free protocols with sub-100 CFU/mL LODs, the demands of real-world food matrices—where target organisms may be present at <1 CFU/25 g, injured by food processing stressors, or embedded within complex fat/protein/salt backgrounds—make direct detection without enrichment exceptionally challenging to validate at the regulatory level. Consequently, the commercial standard remains “enrichment + rapid immunological readout”, with the LFA serving to accelerate the detection step within an otherwise conventional microbiological workflow.

### 5.2. The Translational Gap: Why Most Research-Stage LFAs Do Not Reach the Market

Despite thousands of published research papers annually describing novel LFA configurations with improved sensitivity, multiplexing capability, or innovative signal transduction, the number of LFAs that successfully traverse the full translational pathway to commercial availability remains small. The literature-to-market gap is driven by several well-recognized bottlenecks, listed in Table 6.

### 5.3. Factors Determining Clinical Translation Success

Based on analysis of both successful and failed translational efforts, we identify the following interconnected factors as critical determinants of whether a promising research-stage LFA can be used in practice.

#### 5.3.1. Clinical and Field Validation

The most fundamental requirement for translation is robust validation in clinically and practically relevant settings, which differs substantially from the idealized conditions typical of a proof-of-concept publication. Key elements include the following:

Sample size and diversity: Published studies typically test 3–10 spiked samples per condition. Regulatory validation (e.g., AOAC Performance Tested Methods℠, ISO 16140 series) requires testing across a minimum of 15–30 target strains (inclusivity panel) and 30+ non-target strains (exclusivity panel), often across multiple food types, at multiple contamination levels, and with independent lot replicates. For ISO 16140-2 validation, an inter-laboratory study with 8–15 collaborating laboratories is required.

Real vs. spiked samples: Naturally contaminated samples—where organisms are stressed, injured, and present alongside competitive microflora—behave very differently from laboratory-spiked samples. Many LFAs that perform excellently with fresh overnight cultures lose >90% sensitivity when challenged with starved, cold-stressed, or acid-adapted cells typical of real food processing environments.

User variability: LFA readout in research settings is often performed by the developer with deep familiarity with the assay. In field settings, operators with minimal training must achieve equivalent results. Visual readout at near-LOD concentrations is particularly susceptible to inter-operator variability (±20–40% in some studies), driving interest in instrumented readout for commercial products.

#### 5.3.2. Manufacturing Consistency

Manufacturing reproducibility is arguably the most underappreciated barrier in the LFA translational literature. A research laboratory producing 50–200 strips by hand-dispensing capture antibodies with a pipette, drying strips in a benchtop desiccator, and hand-assembling conjugate pads generates products that cannot be directly replicated at manufacturing scale. Critical manufacturing challenges include Table 7.

The COVID pandemic provided the most visible example of manufacturing consistency challenges: in the first year of the pandemic, multiple manufacturers experienced lot-to-lot sensitivity variations of 2- to 10-fold in their rapid antigen LFAs, attributable to inconsistent antibody conjugation, membrane variability, and inadequate quality control under emergency-use conditions.

#### 5.3.3. Shelf Life and Stability

A shelf life of at least 12–18 months at ambient temperature (typically 4–30 °C) is the commercial expectation for LFAs marketed to the food industry or point-of-care settings. This requirement imposes constraints that are largely absent from research-stage work:

Antibody stability on nitrocellulose: Antibodies dried onto nitrocellulose membranes undergo progressive loss of antigen-binding activity over time due to hydrophobic surface-induced denaturation, oxidation, and aggregation. Stabilizers (e.g., trehalose, sucrose, BSA, PVP) partially mitigate this, but formulation optimization for long-term stability is empirically determined and specific to each antibody-membrane combination.

Conjugate stability: Colloidal gold nanoparticles are prone to aggregation over time, particularly in the presence of salt or upon freeze–thaw stress. Fluorescent labels (quantum dots, europium nanoparticles, organic fluorophores) offer distinct spectral advantages but generally exhibit more rapid photobleaching and require protection from light throughout shelf life.

Accelerated stability testing: Industry-standard protocols (e.g., storage at 37 °C for 30 days, 45 °C for 14 days, or 55 °C for 7 days) are used to predict real-time stability. However, the Arrhenius model does not always hold for complex biological reagents, and real-time stability data remain essential for regulatory submission. A minimum of 12 months of real-time data is typically required for IVD registration in major markets (FDA 510(k), CE-IVDR).

Cold chain requirements: LFAs requiring refrigerated storage (2–8 °C) lose significant field applicability for food safety and low-resource settings where cold chain infrastructure is unreliable. Ambient-temperature stability is therefore a de facto requirement for commercial food-safety LFAs, and this has been a major barrier for nucleic acid lateral flow assays (NALFA) that incorporate enzymes (e.g., horseradish peroxidase for signal amplification), which are inherently less stable at ambient temperature.

#### 5.3.4. Regulatory Requirements

The regulatory pathway for an LFA depends on its intended use, geographic market, and whether it is positioned as a screening or confirmatory method. The following summarizes key regulatory frameworks in Table 8.

Practical considerations: AOAC-RI PTM certification has become the de facto global standard for commercial food pathogen LFAs (Neogen, Merck, Romer Labs, Hygiena, and Charm Sciences products listed in Table 5 all carry this certification). The certification requires a minimum of an independent laboratory study and an inter-laboratory study, with all data reviewed by an AOAC expert reviewer panel. The cost (typically USD 80,000–150,000 per method) and time required (12–24 months from protocol approval to certification) represent a significant barrier for academic research groups.

Enrichment and regulatory realism: Regulatory bodies universally treat LFA results as presumptive positive screening results requiring cultural or molecular confirmation. This fundamental positioning—that LFAs screen, not confirm—shapes the commercial viability of any given LFA: incremental sensitivity improvements below what enrichment already provides (≤10 CFU/mL) may not confer commercial advantage if the method still requires cultural confirmation for regulatory action.

China-specific developments: China’s National Medical Products Administration (NMPA) has introduced expedited review pathways for innovative IVD products, and SAMR’s 2026 release of 13 standardized colloidal gold methods for food safety screening signals growing institutional acceptance. However, LFA methods for foodborne pathogens in China remain primarily limited to laboratory-based screening by government testing agencies, with relatively limited adoption by food producers for in-process testing, in contrast to the more mature commercial adoption in the US and EU.

#### 5.3.5. Economic and Market Factors

Ultimately, technical performance is necessary but not sufficient for commercial translation. Market factors include:

Cost per test: Research LFAs using SERS nanotags, quantum dots, or upconverting nanoparticles may achieve superior LODs, but the incremental cost (SERS tags: USD 3–8 per test in materials alone vs. <USD 1 per test for colloidal gold) limits their market to high-value applications. For routine food safety screening, commercial colloidal gold LFAs are priced at approximately USD 5–15 per test at the end-user level, with manufacturer production costs of USD 0.50–2.00 per strip.

Reader/instrument requirement: LFAs requiring a dedicated fluorescence reader, Raman spectrometer, or magnetic detector incur capital equipment costs (USD 2000–50,000) that limit adoption at small and medium-sized food processing facilities. This has been a significant factor in the dominance of visual-read colloidal gold LFAs, despite their inferior analytical sensitivity.

Throughput and end-user workflow: Food testing laboratories process tens to hundreds of samples per day. LFAs that require complex multi-step protocols (e.g., separate nanotag incubation, wash steps, substrate addition) are incompatible with high-throughput workflows unless integrated into automated systems.

## 6. Conclusions

This review provides an in-depth analysis of the use of LFA for detecting pathogens. It covers the foundational concepts of LFAs and highlights the notable progress that has been made to optimize and simplify the technology in recent years. The evolution of reporters for target labeling has been thoroughly discussed. The review emphasizes how modifications in reporter molecules and the incorporation of signal enhancement techniques have led to greater flexibility and diversity in LFAs. These advancements have not only improved sensitivity and reproducibility but have also helped to lower the costs associated with production. Furthermore, the review delves into the practical applications of LFAs in detecting a range of viruses and bacteria, using specific examples to illustrate their utility. The continuous refinement of LFA technology is a testament to the persistent efforts of researchers in the field. Ongoing research is not only sustaining the progress of LFAs but also expanding their potential in diagnostic testing.

The widespread use of rapid antigen tests for COVID has demonstrated the potential of LFA technology to deliver both high sensitivity and specificity. The simplicity and efficiency of these tests, providing rapid and accurate results, have been crucial for point-of-care testing by the general public during the pandemic, underscoring their vital role in curbing disease spread. As we look to the future, the integration of digital technologies, such as smartphones and wireless communication platforms, is expected to further enhance diagnostic capabilities. This fusion of lateral flow assays with digital innovations presents promising opportunities for disease control and health management. By harnessing the power of digital connectivity, LFAs can become even more accessible and informative, revolutionizing the way we approach diagnostics and healthcare.

While the integration of advanced nanoreporters has undeniably pushed the limits of detection in lateral flow assays, the path toward widespread clinical adoption remains obstructed by significant engineering and economic challenges. The complexity of synthesizing anisotropic nanomaterials often results in poor batch-to-batch reproducibility, creating a barrier to the standardization required for regulatory approval. Furthermore, the long-term stability of these conjugates—specifically their resistance to aggregation and oxidation under varying storage conditions—remains a critical unresolved issue. Future research must therefore pivot from purely maximizing sensitivity to addressing these ‘real-world’ constraints, focusing on robust, scalable manufacturing protocols that ensure cost-effectiveness and reliability comparable to traditional colloidal gold standards.

## Figures and Tables

**Table 1:** Comparison of effect of label size on the LFA.

Factor	Effect of Smaller Label	Effect of Larger Label
Diffusion rate	Faster diffusion → faster binding kinetics → more label captured per unit time at test line	Slower diffusion → potentially slower kinetics
Membrane transport	Easier penetration through nitrocellulose pore network; less steric entrapment	May experience restricted mobility at pore constrictions; potential for nonspecific entrapment
Signal per binding event	Lower—fewer fluorophores, less chromophore mass, less catalytic capacity per label	Higher—more reporter payload per label → stronger signal at each captured site
Steric hindrance	Lower—less interference with antigen–antibody binding	Higher—may block adjacent epitopes or reduce antibody accessibility
Binding valency	Typically lower (fewer antibodies per particle)	Potentially higher (multivalent binding)—can enhance avidity but also increase nonspecific binding

**Table 2:** Virus detection with LFAs.

Viruses	Subtypes	Labels or the Signal Enhancement Methods	Detection Object	Recognition Element	Read-Out Way	Reference
Influenza Virus	H1N1 and H3N2	Carbon nanoparticles	Influenza A nucleoprotein	Antibody	Naked eyes	[118]
	H1N1/HAdV	SERS (Fe_3_O_4_@Ag)	Virus	Antibody	Naked eyes/device	[119]
	H1N1	Entropy-driven amplification /fluorescent nanospheres	RNA	DNA	Device	[122]
	pH1N1	SERS (MGITC)	pH1N1 H275Y neuraminidase	6E3 antibody	Naked eyes/device	[167]
	H3N2	SERS/magnetic (Au NPs@4MBA and Fe_3_O_4_/Au NPs)	Antigen	Antibody	Device	[5]
	H3N2	Gold Nanoparticles	RNA	Aptamer	Naked eyes/device	[123]
HIV	HIV-1	SERS (MGITC)	DNA	DNA	Naked eyes /device	[137]
		Quantum dots	DNA	Hairpin DNA	Device	[11]
	HIV-1/-2	UCNP	Anti-HIV-1/2 antibodies	r-HIV-1env and r-HIV-2 Env antigens	Device	[138]
Coronavirus		Catalytic hairpin assembly reaction	RNA	DNA probes	Device	[112]
		UCNPs@mSiO_2_	Spike protein (SP) and nucleocapsid protein (NP)	Antibodies	Device	[168]
		Red cellulose nanobeads	SARS-CoV-2 spike 1 (S1) protein	ACE2 and antibody	Device	[110]
Ebola		Fe_3_O_4_ magnetic nanoparticle	Glycoprotein	Antibody	Naked eyes	[145]
		Colloidal gold	Glycoprotein and nucleoprotein	Antibody	Naked eyes/device	[169]
		Quantum dots (RNs@Au)	Glycoprotein	Antibody	Naked eyes/device	[146]
Dengue virus		Near-infrared fluorescent dye (DyLight-800)	Anti-DENV1 IgG antibodies	Recombinant dengue type 1 envelope protein	Device	[7]
		PEG-stabilized and TEMPO-tagged AuNPs	Dengue NS1 protein	Antibody	Device	[152]
		Reverse transcription RPA	RNA	/	Naked eyes	[155]
		Gold-decorated graphene oxide (Au-rGO)	Dengue NS1 protein	Antibody	Device	[153]
HPV		Recombinase polymerase amplification (RPA)	Circulating cell-free DNA	DNA	Device	[170]
		CuO NPs	DNA	Capture DNA	Naked eyes/device	[142]
Hepatitis B virus		Quantum dot-beads	Hepatitis B virus surface antigen (HBsAg)	Antibody	Device	[12,95]
		Europium nanoparticle	Hepatitis B core antigen	Antibody	Device	[126]
Hepatitis C Virus		Ag-Ab Combo	HCV core antigen and anti-HCV antibody	Anti-HCV core antibody and combined HCV antigen	Device	[131]
Zika virus		Quantum dots	ZIKV nonstructural protein 1 (NS1)	Antibody	Device	[165]
		Colloidal gold	ZIKV envelope (E) protein	mAbs (A1, B1, C1, and 9E-1)	Device	[166]
		Reverse transcription loop-mediated isothermal amplification (RT-LAMP)	RNA	AuNP: polyadenylated (polyA10) ZIKV probe	Naked eyes/device	[158]

**Table 3:** Detection targets and labels of LFAs for common pathogenic bacteria.

	Labels or the Signal Enhancement Methods	Recognition Element	Read-Out Way	Reference
*Treponema pallidum*	Quantum dot bead	TP15 and TP17Antibody	Device	[106]
*Escherichia coli* O157: H7	Colloidal gold	Antibody	Naked eyes	[108]
	SERS (gold-shell silica-core (SiO2/Au) nanosphere)	Antibody	Device	[109]
	UCNPs (Cucurbit [7] uril-based hydrophilic modified NaYF_4_:2%Er^3+^,18%Yb^3+^@NaYF_4_)	Antibody	Device	[110]
	SERS (DTNB/magnetic Fe_3_O_4_/Au-PEI nanoparticles)	Antibody	Device	[111]
	Functional nanozyme (mannose-modified Prussian blue)	Antibody	Naked eyes/Device	[112]
*L. monocytogenes*	AEDR-AuNPs	Antibody	Naked eyes	[117]
	SERS (4-MBA modified AuNPs)	Antibody	Device	[116]
	Silica nanoparticles	Aptamer	Naked eyes	[115]

**Table 4:** Other detection details and complements are classified according to reporter molecules of detection.

Reporters & Signal Enhancement Techniques		Applications	Recognition Element	Detection Format	Liner Range	Limit of Detection (LOD)	Reference
Nucleic acid	Entropy-driven amplification	H1N1	DNA	Sandwich	NM	2.02 pM	[48]
	SELEX strategies	H3N2	Aptamer	Sandwich	NM	2 × 10^6^ virus particles	[52]
	Catalytic hairpin assembly reaction	SARS-CoV-2	DNA	Sandwich		2000 copies/mL	[68]
	Reverse transcription RPA	Dengue fever	/	Sandwich	/	10 copies/μL	[79]
	Recombinase polymerase amplification	HPV	DNA	Sandwich		80 copies/mL HPV16 E7 DNA, 86 copies/mL HPV18 E7 DNA	[85]
	RT-LAMP	ZIKV	/	Competitive	3–3 × 10^5^ copies	Lower than 3 copies	[103]
	Aptamer-gated silica Nanoparticles	*Listeria monocytogenes*	Aptamer	/		53 cells/mL	[116]
Near-infrared dyes	DyLight-800	Dengue fever	Antibody	Sandwich			[17]
Gold particles	-	Dengue fever	Antibody	Sandwich	100 to 1000 ng/mL	50 ng/mL	[78]
	Au-rGO reduced graphene oxide	Dengue fever	Antibody	Sandwich	3 to 25 ng/mL	4.9 ng/mL	[82]
	Colloidal gold	ZIKV	Antibody	Sandwich		6.3 × 10^6^ pfu/mL	[102]
	Colloidal gold	*Escherichia coli* O157: H7	Antibody	Sandwich		O157: 10^3^ CFU/mL and H7: 10^4^ CFU/mL	[108]
	AEDR-AuNPs	*Listeria monocytogenes*	Antibody	Sandwich		2.5 × 10^5^ CFU/mL	[117]
Magnetic particles	MnFe_2_O_4_	H1N1	Antibody	Sandwich		0.007 HAU	[39]
	Fe_3_O_4_ magnetic nanoparticle	Ebola virus	Antibody	Sandwich		1 ng/mL	[72]
	Fe3O4/Au-PEI (SERS)	*Escherichia coli* O157: H7	Antibody	Sandwich	10^1^–10^7^ CFU/mL	0.52 CFU/mL	[111]
Quantum dots	-	HBV	Antibody	Sandwich	75 pg/mL to 75 ng/mL	75 pg/mL	[23]
	-	HIV-1	Hairpin DNA	Sandwich	1 pM to 10 nM	0.76 pM	[59]
	-	Ebola virus	Antibody	Sandwich	2–1000 ng/mL	0.18 ng/mL	[74]
	-	HBV	Antibody	Sandwich	0.05–3200 ng/mL	50.0 pg/m	[90]
	Carboxyl-functionalized CdSe/ZnS	ZIKV	Antibody	Sandwich		0.045 ng mL	[101]
	-	*Treponema pallidum*	Antibody	Sandwich		0.62 IU/mL	[106]
Fe_3_O_4_@Ag	-		Antibody	Sandwich	10^2^ to 5 × 10^3^ TCID_50_/mL	10^2^ TCID_50_/mL	[14]
	-	H1N1, H3N2	Antibody	Sandwich		350 TCID_50_/mL	[45]
	Fe_3_O_4_@Ag	H1N1/HAdV	Antibody	Sandwich	10–10^7^pfu /mL	50 and 10 pfu/mL	[46]
	-	pH1N1	Antibody	Sandwich		10^2^ PFU/mL	[51]
	-	HIV-1	DNA	Sandwich	8 pg/mL to 64 ng/mL	0.24 pg/mL	[58]
	SiO_2_/Au	*Escherichia coli* O157: H7	Antibody	Sandwich		50 cells/mL	[109]
	4-MBA-conjugated AuNPs	*Listeria monocytogenes*	Antibody	Sandwich	10^2^–10^7^ CFU /mL	75 CFU/mL	[115]
Carbon nanoparticles		HIV-1/-2	Antibody	Sandwich			[60]
Upconversion nanoparticles	-	SARS-CoV-2	Antibody	Sandwich		1.6 ng/mL/ 2.2 ng/mL	[70]
	-	HPV	DNA	Sandwich	5 nM to 100 nM	1.0 nM	[86]
	UCNPs-PAA-Ab	*Escherichia coli* O157: H7	Antibody	Sandwich	5 × 10^4^–1 × 10^7^ CFU/mL	2.8 × 10^4^ CFU/mL	[110]
CuO	-	HPV	DNA	Sandwich	5 nM to 100 nM	1.0 nM	[86]
Sliver enhancement	Ag-Ab Combo	HCV	Anti-HCV core antibody and combined HCV antigen	Sandwich			[94]
Europium nanoparticle	-	HBV	Antibody	Sandwich	0.63–640 IU/mL	0.31 IU/mL	[91]

Abbreviations: EID50: 50% Egg Infective Dose, HAU: hemagglutinin units, CFU: colony-forming units, IU: international units, PFU: plaque-forming units, SERS: surface-enhanced Raman scattering, TCID50: tissue culture infection dose at 50% endpoint. Conclusion and outlook.

**Table 5:** Commercially available LFAs.

Manufacturer	Product/Platform	Target (s)	Format	Validation	Approximate Time to Result
Neogen (formerly 3M Food Safety)	Reveal^®^ 2.0	*Salmonella*, *Listeria* spp., *L. monocytogenes*, *E. coli* O157:H7	Sandwich LFA (colloidal gold)	AOAC-RI PTM certified. AOAC International: Rockville, MD, USA, 2023.	15–20 min (after enrichment: 15–48 h)
Merck/MilliporeSigma	Singlepath^®^/Duopath^®^	*Salmonella*, *Listeria*, *E. coli* O157:H7, *Campylobacter*	Sandwich LFA	AOAC-RI AOAC International: Rockville, MD, USA, 2023., AFNOR La Plaine Saint-Denis, France, 2021 validated.	20 min (after enrichment)
Bio-Rad	RAPID’Chromogenic media + confirmation	*Listeria* spp., *Salmonella*	Combination culture + immunological	ISO 16140-3:2021, Microbiology of the food chain — Method validation, Geneva, Switzerland, 2021	Culture-dependent
Romer Labs (DSM)	RapidChek^®^	*Salmonella*, *Listeria*	Sandwich LFA with proprietary phage-based enrichment	AOAC-RI PTM. AOAC International: Rockville, MD, USA, 2023.	10 min (after enrichment: 12–44 h)
Hygiena	InSite™	*Salmonella*, *Listeria*, *E. coli*	LFA integrated with environmental swabs	AOAC-RI, AOAC International: Rockville, MD, USA, 2023.	15–30 min
Charm Sciences	Charm^®^ ROSA	*Salmonella*, *Listeria*, *E. coli* O157:H7	Receptor-based lateral flow	AOAC-RI, AOAC International: Rockville, MD, USA, 2023.; AFNOR, La Plaine Saint-Denis, France, 2021.	10–20 min
BioAssay Works	ETLF-020	*E. coli* O157:H7	Sandwich LFA (colloidal gold)	Research-use only	15 min

**Table 6:** Key barriers in translating research-stage LFAs to commercial products.

Stage	Common Failure Point	Contributing Factors
Antibody/Receptor Selection	Antibodies selected on purified antigen fail on whole-cell targets or real matrices	Lack of testing on stressed/injured cells; epitope masking by food matrix components
Prototype → Pilot Scale	Performance degrades upon scale-up from hand-assembled strips (*n* = 50–100) to pilot manufacturing (*n* = 10,000+)	Membrane lot-to-lot variability; inconsistent antibody conjugation efficiency; drying/humidity sensitivity
Validation	Sensitivity/specificity established in spiked buffer does not generalize	Matrix effects (fat, protein, pH, salt, competitive flora); target strain diversity not accounted for
Stability/Shelf Life	Signal degrades after weeks at ambient storage	Colloidal gold aggregation; antibody denaturation on nitrocellulose; nitrocellulose hydrophobic recovery
Regulatory	Insufficient data package for AOAC/ISO/FDA submission	Inclusivity/exclusivity panel too small; no inter-laboratory study; no lot-to-lot consistency data

**Table 7:** Critical manufacturing challenges in LFAs.

Parameter	Research Scale	Manufacturing Scale	Key Challenge
Antibody dispensing	Manual pipetting or single-channel dispenser (CV 10–15%)	Automated reel-to-reel dispensing (CV < 3% required)	Line uniformity across the full membrane width; edge effects
Conjugate preparation	Small-batch conjugation (1–10 mL); OD measured by spectrophotometer	Large-batch conjugation (100–500 mL); requires consistent particle size, antibody loading density, blocking efficiency	Batch-to-batch OD and functional activity consistency
Drying	Ambient or benchtop oven (uncontrolled humidity)	Controlled-environment tunnel drying (temperature, humidity, air flow precisely regulated)	Over-drying causes antibody denaturation; under-drying causes flow inconsistency
Membrane lot variability	Single lot typically sufficient for all experiments	Must qualify and blend membrane lots to maintain consistent flow rate and binding capacity	Nitrocellulose pore structure is inherently variable; polymer aging alters surface properties
Lamination and Cutting	Hand assembly; guillotine or rotary cutter	Automated lamination and high-speed cutting (±0.1 mm tolerance)	Component overlap/alignment affects flow dynamics; cutting pressure affects edge wicking
QC Lot Release	Visual inspection of test line	Quantitative line intensity (CV <10% at target concentration); accelerated stability testing (e.g., 7 days at 55 °C equivalent to ~12 months at RT)	Requires dedicated QC strips sacrificed from each lot

**Table 8:** Key regulatory frameworks.

Jurisdiction	Applicable Framework	Key Requirements for LFA Validation
USA (FDA)	510(k) premarket notification (Class II medical device) or De Novo classification	Analytical sensitivity/specificity; clinical agreement study (prospective, ≥30 positive specimens); precision (repeatability/reproducibility across 3 sites × 5 days × 2 operators × 2 lots); stability (real-time + accelerated); labeling
USA (AOAC-RI)	Performance Tested Methods℠ (PTM)—for food testing methods, not medical devices	Inclusivity (≥30 strains), exclusivity (≥30 non-target organisms), matrix study (1–3 food types), robustness (minor variations in incubation time/temperature), lot-to-lot consistency (3 lots), independent laboratory verification
EU (CE-IVDR)	In Vitro Diagnostic Medical Devices Regulation (EU 2017/746)	Classification-based requirements (Class A–D); notified body review for Class B–D; clinical evidence per Annex XIII; post-market surveillance plan
EU (ISO 16140)	Microbiology of the food chain—Method validation	ISO 16140-2: validation against reference method; ISO 16140-4: single-laboratory validation; ISO 16140-6: microbiological confirmation procedure validation
China (NMPA)	Medical Device Registration (Class II or III)	Product technical requirements, type testing at accredited laboratory, clinical trial or clinical evaluation, quality management system audit (GMP)
China (SAMR)	Food rapid detection method evaluation (per Food Safety Law Art. 112)	Method performance verification against GB standard methods; inter-laboratory comparison; intended for screening only

## Data Availability

The data presented in this review are available in the cited original publicly accessible journals.
